# Transcription-Factor-Mediated DNA Looping Probed by High-Resolution, Single-Molecule Imaging in Live *E. coli* Cells

**DOI:** 10.1371/journal.pbio.1001591

**Published:** 2013-06-18

**Authors:** Zach Hensel, Xiaoli Weng, Arvin Cesar Lagda, Jie Xiao

**Affiliations:** Department of Biophysics and Biophysical Chemistry, Johns Hopkins University School of Medicine, Baltimore, Maryland, United States of America; National Cancer Institute, United States of America

## Abstract

DNA looping mediated by transcription factors plays critical roles in prokaryotic gene regulation. The “genetic switch” of bacteriophage λ determines whether a prophage stays incorporated in the *E. coli* chromosome or enters the lytic cycle of phage propagation and cell lysis. Past studies have shown that long-range DNA interactions between the operator sequences *O_R_* and *O_L_* (separated by 2.3 kb), mediated by the λ repressor CI (accession number P03034), play key roles in regulating the λ switch. In vitro, it was demonstrated that DNA segments harboring the operator sequences formed loops in the presence of CI, but CI-mediated DNA looping has not been directly visualized in vivo, hindering a deep understanding of the corresponding dynamics in realistic cellular environments. We report a high-resolution, single-molecule imaging method to probe CI-mediated DNA looping in live *E. coli* cells. We labeled two DNA loci with differently colored fluorescent fusion proteins and tracked their separations in real time with ∼40 nm accuracy, enabling the first direct analysis of transcription-factor-mediated DNA looping in live cells. Combining looping measurements with measurements of CI expression levels in different operator mutants, we show quantitatively that DNA looping activates transcription and enhances repression. Further, we estimated the upper bound of the rate of conformational change from the unlooped to the looped state, and discuss how chromosome compaction may impact looping kinetics. Our results provide insights into transcription-factor-mediated DNA looping in a variety of operator and CI mutant backgrounds in vivo, and our methodology can be applied to a broad range of questions regarding chromosome conformations in prokaryotes and higher organisms.

## Abstract 2

A high-resolution, single-molecule study directly assesses the prevalence and dynamics of DNA looping in gene regulation in live E. coli cells.

## Author Summary

One mechanism cells use to regulate gene expression is DNA looping, whereby two distant DNA sites are brought together by regulatory proteins. The looping then either enhances interactions between other regulatory proteins bound at the separate sites or brings those regulatory proteins close to RNA polymerase at the promoter. Recent work in bacteriophage λ has suggested that DNA looping mediated by a transcription factor called λ repressor CI plays a critical role in regulating the expression of λ genes and consequently in determining the fate of the host *E. coli* bacterial cells. CI-mediated DNA looping has been directly demonstrated in vitro, but it has only been indirectly inferred in vivo. For the current study we developed a method to visualize CI-mediated DNA looping in individual live *E. coli* cells. We labeled two DNA sites—one each side of the proposed loop—with differently colored fluorescent fusion proteins, allowing us to measure their separation with an accuracy of a few tens of nanometers. Using this method, we directly analyzed CI-mediated DNA looping, providing insight into how transcription factor-mediated DNA looping influences gene regulation in live *E. coli* cells. Our methodology can be applied to a broad range of questions regarding chromosome conformation in prokaryotes and higher organisms.

## Introduction

Looping between two DNA sites, mediated by transcription factors, is a ubiquitous mechanism in prokaryotic transcription regulation [1]. DNA looping brings two distal DNA sites into close proximity, enhancing interactions between transcription factors bound at separate sites or bringing transcription factors close to RNA polymerase at the promoter. Knowing when and how DNA loops in vivo is important to understand the role of DNA looping in gene regulation and cell decision-making; some studies found molecular details of gene regulation have little influence on gene expression [2]–[4], while others suggested that DNA looping could trigger cell phenotype switching [5] and influence fluctuations in transcription activity [6].

DNA looping was first suggested for the transcription factor AraC (accession number P0A9E0) in the *E. coli* arabinose operon. Disruption of an AraC binding site ∼280 bp upstream of the promoter reduced AraC-mediated repression nearly 10-fold, indicating a long-range interaction between the promoter and upstream DNA [7]. Subsequently, DNA looping mediated by transcription factors LacI [8] (accession number P03023), DeoR [9] (accession number P0ACK5), NtrC [10] (accession number P0AFB8), GalR [11] (accession number P03024), and bacteriophage λ repressor CI [12],[13] was reported. The length of the intervening DNA in these loops can be as short as 58 bp (*lac* operon [8]) or as long as ∼5 kilobases (*deo* operon [9]).

Biochemical, biophysical, and genetic studies have established important roles of DNA looping in transcription regulation. However, transcription-factor-mediated DNA looping on the length scale of a few kilobases in prokaryotic cells has not been directly visualized in vivo, and the in vivo dynamics of DNA looping are difficult to investigate. Chromosome conformation capture (3C) has been used to detect juxtaposition of DNA sites separated by hundreds of kilobases in both eukaryotic and prokaryotic cells [14],[15], but high background of interactions at the kilobase scale limits the utility of these methods in studying typical prokaryotic DNA loops [16]. An in vivo imaging method using fluorescent proteins fused to DNA-binding proteins bound to tandem arrays of hundreds of binding sites has been employed to visualize homologous chromosome pairing in yeast induced by double-strand breaks [17]; however, an array of several kilobases of binding sites makes this method unsuitable for studying DNA loops of only a few kilobases. In addition, the long array of tightly bound protein molecules may be detrimental to cells [18].

**Figure 1:**
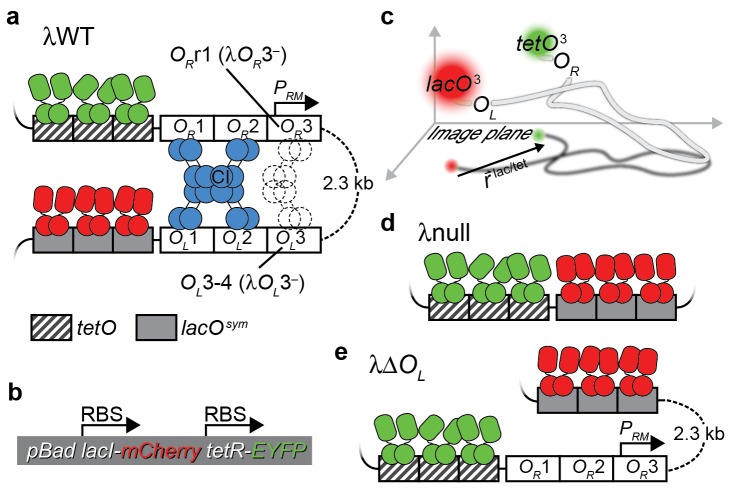
Visualizing DNA looping in vivo by localizing *O_R_* and *O_L_* with fluorescent DNA-binding fusion proteins. (a) λWT construct. Three tandem *lacO^sym^* and *tetO* sites, termed *lacO^3^* and *tetO^3^*, were placed immediately next to *O_L_* and *O_R_*, respectively. Red and yellow fluorescent fusion proteins LacI-mCherry and TetR-EYFP bind *lacO^3^* and *tetO^3^*, respectively. DNA looping mediated by a CI octamer (blue) or an additional CI tetramer (dashed) brings *lacO^3^* and *tetO^3^* together. Strains λ*O_R_*3^−^ and λ*O_L_*3^−^ harbor mutations (described in main text) to *O_R_*3 and *O_L_*3, respectively, that prevent CI dimers from binding these operator sites. (b) LacI-mCherry and TetR-EYFP are expressed co-transcriptionally from separate ribosome binding sites on a plasmid. (c) Illustration of 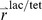 measurement. The observed distance between mCherry and EYFP spots indicates the distance between *lacO^3^* and *tetO^3^* projected onto the imaging plane. (d) Positive control λnull. The centers of *lacO^3^* and *tetO^3^* are separated by only 66 bp (see Figure S2a). (e) Negative control λΔ*O_L_*. *O_L_* is deleted to eliminate CI-mediated DNA looping.

We developed a two-color, high-resolution imaging method to directly measure the end-to-end separation of two DNA sites 2.3 kb apart in live *E. coli* cells (Figure 1a). This method is based on the ability to precisely determine the location of a specific DNA site in vivo [19]. By expressing a fluorescent protein in fusion with a DNA-binding protein in a cell with only three tandem binding sites (spanning less than 100 bp), the resulting fluorescent spot is diffraction-limited, and the location of the binding site can be determined with sub-diffraction-limited precision by fitting its fluorescence profile to a two-dimensional Gaussian function [20]. By labeling two ends of a DNA segment with two unique sets of binding sequences and co-expressing corresponding fluorescent DNA-binding fusion proteins of different colors, the distance between the two DNA sites can be determined with a precision of a few tens of nanometers. An in vitro experiment employing the same principle measured intramolecular distances using organic dyes [21], but this approach has not been demonstrated in vivo with comparable resolution using fluorescent proteins.

We used our method to probe the mechanisms and dynamics of DNA looping mediated by the bacteriophage λ repressor CI [22] in live *E. coli* cells and investigate its regulation of transcription from the CI promoter *P_RM_*. The λ repressor CI is an essential transcription factor in determining the fate of an *E. coli* cell infected by the bacteriophage λ. When CI is expressed, it represses lytic promoters to commit to an extraordinarily stable lysogenic state that persists for millions of generations [23]–[25]. However, upon induction by UV irradiation or other specific events, CI degradation can trigger an irreversible switch from lysogenic to lytic gene expression within one cell generation time [26].

The robustness of the λ regulatory circuit has been extensively studied. Among many important features of the system such as promoter-operator arrangement [27],[28], CI autoregulation [3],[29],[30], and cooperative binding [31]–[34], DNA looping between the homologous rightward and leftward operators *O_R_* and *O_L_*, separated by 2.3 kb, was shown to play significant, fate-determining roles in the λ lifecycle [13],[35]. Cooperative binding of CI dimers at the subsites *O_R_*1 and *O_R_*2 of *O_R_* represses the lytic promoter *P_R_* (reviewed in [36]) and simultaneously activates CI's own promoter, *P_RM_*, by accelerating transcription initiation [37]–[39]. At higher CI concentrations, an additional CI dimer binds to *O_R_*3 and represses *P_RM_* [40].

As illustrated in Figure 1a, an octameric CI complex (with or without an additional CI tetramer) can mediate DNA looping by bridging *O_R_* and *O_L_*. These higher-order complexes result from interactions between CI dimers bound to subsites at *O_R_123* and *O_L_123*, and were first identified in vitro by ultracentrifugation [41] and later visualized by EM [12] and AFM [42]. Looping dynamics were investigated in vitro using tethered particle motion (TPM) [43]–[46].

To gain quantitative insight into the relationship between CI-mediated DNA looping and transcription regulation, thermodynamic models and numerical simulations were developed [33],[35],[44],[47]–[52]. Key parameters in these studies were the free energies of octameric and tetrameric CI interactions that mediate DNA looping [35]. These free energies specify the DNA looping probability at a given condition (temperature, CI concentration, etc.) and hence the extent to which distal DNA sites affect each other. To date, DNA-looping probabilities and free energies were either estimated indirectly in in vivo studies by measuring *P_RM_* and *P_R_* activities in various operator mutants with a priori assumptions of DNA looping states [35],[49],[51] or measured using purified components in vitro, where conditions differ from those in a cellular environment [42]–[46]. Consequently, these studies yielded varying estimates for the free energies of DNA looping and the degree to which DNA looping influences *P_RM_* activity. Hence, the roles of CI-mediated DNA looping in transcription regulation are still in debate [13],[35],[49],[51],[53].

In this study, we tracked the apparent separation between the *O_R_* and *O_L_* sites on a λ DNA segment (termed *O_R_*–*O_L_* DNA below) in real time in live *E. coli* cells, from which we obtained the first direct estimates of in vivo looping frequencies and kinetics for both wild-type DNA and for DNA carrying mutations in *O_R_*3 and *O_L_*3. We also measured corresponding CI expression levels in these strains by counting the number of CI transcripts in individual cells. Applying these independent, in vivo measurements to a thermodynamic model, we were able to obtain looping free energies and quantify the influence of DNA looping on *P_RM_* expression. Furthermore, we discuss how the compaction of the *E. coli* chromosome may impact DNA looping kinetics. The methodology established in this work can be extended to a broad range of questions regarding chromosomal DNA conformation and/or gene activities in prokaryotes and higher organisms.

## Results

### High-Resolution Imaging of Two DNA Sites

We inserted the construct shown in Figure 1a into the *E. coli* chromosome. It contains three tandem *tetO* sites (*tetO^3^*) [54] and three tandem *lacO^sym^* sites (*lacO^3^*) [55] flanking the wild-type λ lysogen sequence from *O_R_* to *O_L_* (including the *P_R_, P_RM_ and P_L_* promoters and the *cI*, *rexA* (accession number P68924) and *rexB* (accession number P03759) genes). In this construct, called λWT, CI is expressed from *P_RM_* and regulates its own expression. The *lacO*-binding and *tetO*-binding proteins LacI and TetR (accession number P04483) were fused with red and yellow fluorescent proteins to generate LacI-mCherry and TetR-EYFP, and were expressed from an inducible plasmid (Figure 1b).

**Figure 2:**
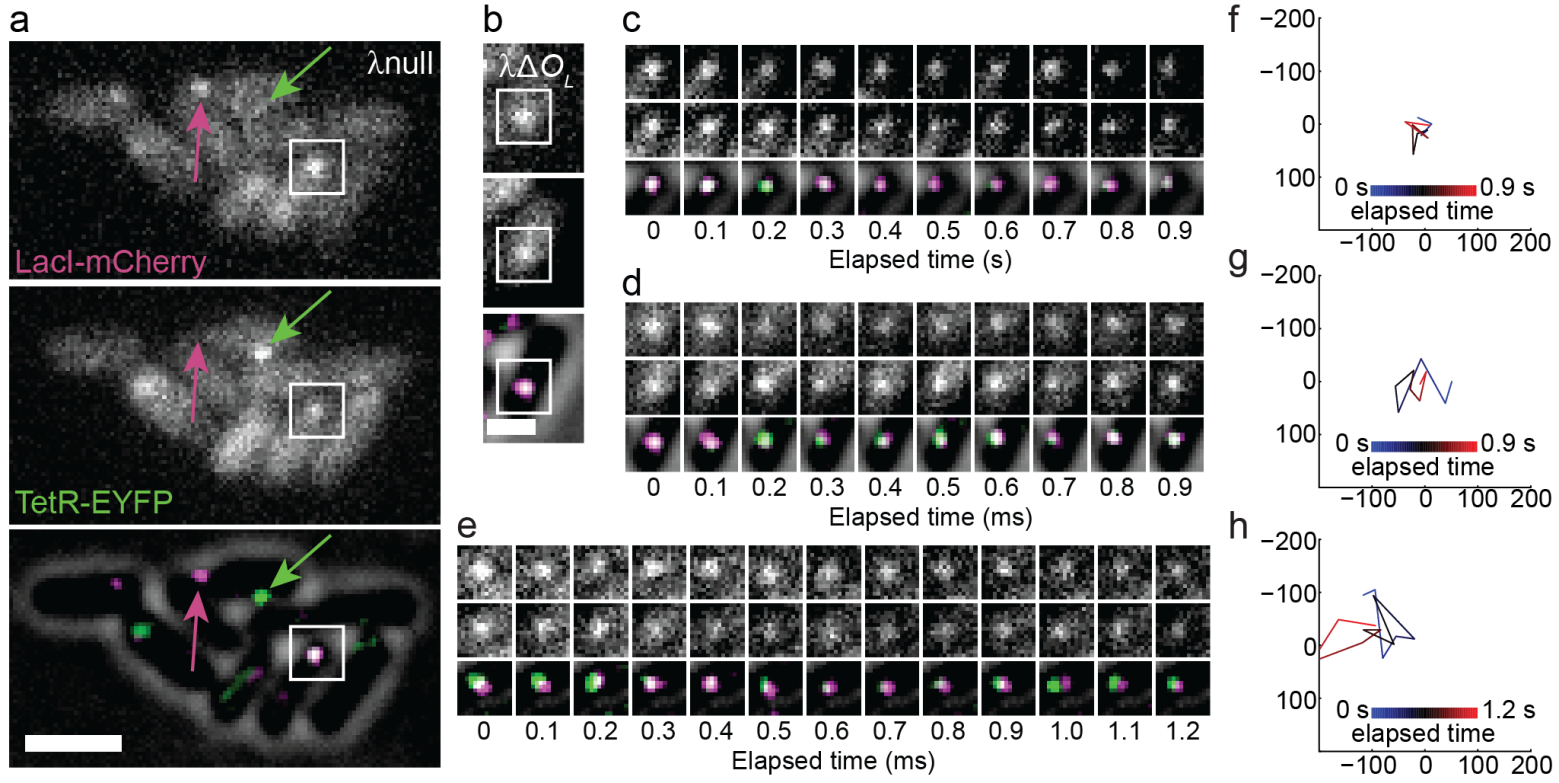
High-resolution imaging of *lacO^3^* and *tetO^3^* sites separated by 66 bp (λnull) or 2.3 kb (λΔ*O_L_*). (a) Fluorescent images of λnull. Arrows highlight molecules that exclusively appeared in mCherry (magenta, top) and EYFP (green, middle) channels, indicating a lack of significant crosstalk between the two channels. Squares show a spot that appeared in both channels. In the overlay image (bottom), fluorescence images were bandpass filtered and background was subtracted. Only cells having both mCherry and EYFP fluorescence were used in analysis. Scale bar, 2 µm. The image order and color scheme are repeated in (b–e). (b) Fluorescent images of λΔ*O_L_*. Scale bar, 1 µm. (c–e) Timelapse images of spots acquired every 100 ms; (c) and (d) are spots in white squares in (a) and (b), respectively, and (e) shows an additional λΔ*O_L_* spot, whose apparent separation can be easily detected by eye. Top and middle rows show mCherry and EYFP channels, respectively, and bottom rows show two-color overlays on brightfield images. (f–h) Trajectory 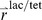 vectors from fitting fluorescence data for spots in (c–e). Coordinates are in nm. Vertices indicate the 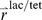 vector and subsequent time points are connected by lines that are colored to indicate elapsed time.

With the combination of strong induction, weak ribosome binding sites, and carefully controlled growth, we achieved sufficiently low LacI-mCherry and TetR-EYFP expression levels to detect distinct, diffraction-limited mCherry and EYFP spots in single cells. We then fit the fluorescence intensity profile of each individual spot with a two-dimensional Gaussian function to estimate its centroid position. The average localization precisions for individual spots of LacI-mCherry and TetR-EYFP were 17 and 14 nm, respectively (Figure S1a). Subsequently, we transformed EYFP coordinates into mCherry coordinates using fiducial data to calculate the vector between the mCherry and EYFP spots arising from LacI-mCherry and TetR-EYFP protein molecules bound to the same *O_R_*–*O_L_* DNA segment. We called this vector 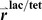 (Figure 1c). The magnitude of the vector, 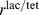, is the two-dimensional projection of the distance between *lacO^3^* and *tetO^3^* onto the image plane; on average, it is proportional to the end-to-end distance between *lacO^3^* and *tetO^3^* in three dimensions. The total error for an 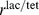 measurement, including fitting errors in determining centroid of individual spots (Figure S1a), registration errors in aligning EYFP and mCherry two-color images (∼10 nm based upon experiments using fluorescent beads), and contributions from local fluorescent background, was on average ∼40 nm (see below). With very low TetR-EYFP and LacI-mCherry expression, it was inevitable that not all *lacO^3^* and *tetO^3^* sites were bound by fusion protein molecules. Furthermore, not all fusion protein molecules were fluorescent due to stochastic chromophore maturation. Figure 2a contains typical data showing that a subset of cells was successfully labeled at both sites. We analyzed all cells having distinct fluorescent spots in both emission channels to calculate 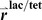. We expected 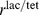 to decrease when DNA between *lacO^3^* and *tetO^3^* looped.

### Distinguishing Between Looped and Unlooped States

To determine whether our two-color imaging method was sufficient to distinguish between looped and unlooped DNA in the crowded intracellular environment, we constructed two control strains (Table 1). In the positive control λnull, the centers of *lacO^3^* and *tetO^3^* sites are separated by 66 bp (Figure 1d). The outmost *lacO^sym^* and *tetO* sites are separated by less than 40 nm (Figure S2a). The close proximity of *lacO^3^* and *tetO^3^* mimicked permanently looped DNA. In the negative control λΔ*O_L_*, we inserted the λ sequence from *O_R_* up to but not including *O_L_* between *lacO^3^* and *tetO^3^* (Figure 1e). The resulting λΔO_L_ DNA has comparable length as the wild-type λ DNA, but CI-mediated DNA looping between *O_R_* and *O_L_* is abolished.

**Table 1:** Descriptions of strains and plasmids used in this study.

Strain Name	Genotype
λnull	MG1655 Δ*lacIzya*::*lacO^3^tetO^3^*
λWT	λnull *lacO^3^tetO^3^*::(*O_R_–O_L_*)
λΔ*O_L_*	λWT Δ*O_L_*
λ*O_R_*3^−^	λWT *O_R_*3r1
λ*O_L_*3^−^	λWT *O_L_*3–4
λΔ*O_L_P_RM_^−^cI^−^*	λΔ*O_L_ P_RM_^−^cI^−^*
λΔ*O_L_P_RM_^−^cI^−^/cI^trans^*	λΔ*O_L_ P_RM_^−^cI^−^* (pACL18)
λCI^G147D^	λWT *cI(G147D)*
λCI^G147D^/*cI^G147D,trans^*	λCI^G147D^ (pACL17)
pZH102R33Y29	pLau53 [82] *pBad*-{*lacI-mCherry*}-{*tetR-EYFP*}
pZH102R33TD	pLau53 *pBad*-{*lacI-mCherry-EYFP*}
pACL18	pACYC184-*cI* ^wt^
pACL17	pACYC184-*cI* ^G147D^

The 2.3-kb, wild-type phage λ sequence from *O_R_* to *O_L_* was incorporated into the *E. coli* chromosome in λWT. Strains λ*O_R_*3^−^, λ*O_L_*3^−^, and λWT^G147D^ contain the *r1* [80], OL3–4 [35], and CI^G147D^ [62] mutations, respectively. Curly brackets indicate fusion products expressed from a single ribosome binding site. These are shorthand strain names; names used in our lab are listed in Table S4.

We first examined λnull and λΔ*O_L_* in two-color fluorescence images to determine whether we could discriminate between looped and unlooped DNA by eye. We obtained at least sixty 20-frame movies (100 ms exposures; 2 s total) for each strain in each of three independent experiments. Typical fluorescence images are shown in Figure 2a and b. Crosstalk between the two emission channels was negligible, as bright mCherry and EYFP spots only appeared in the corresponding channel but not the other.

Figure 2c and d show 1 s of typical data for individual λnull and λΔ*O_L_* spots. Representative movies for the two strains and others discussed below are included as Movies S1, S2, S3, S4, S5, S6. As expected for a permanently looped configuration, the positive control λnull exhibited overlapping EYFP and mCherry spots (Figure 2c). Generally, λΔ*O_L_* molecules did not exhibit spot separation that was easily identifiable by eye (Figure 2d). However, some λΔ*O_L_* molecules displayed large displacements between the LacI-mCherry and TetR-EYFP spots that were distinguishable by eye (Figure 2e); such images were not observed for λnull.

**Figure 3:**
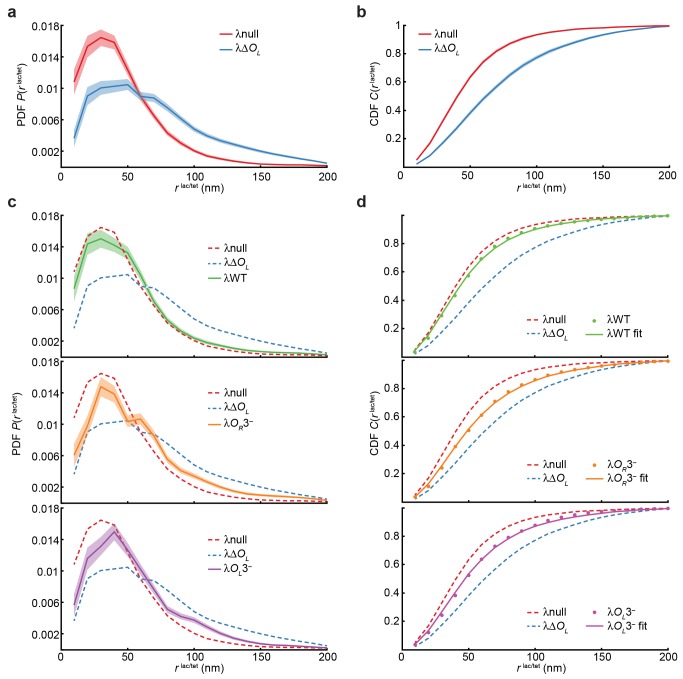
End-to-end distance (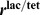) distributions and looping frequency fitting. (a) Probability density distribution (PDF) of the 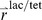 vector magnitude 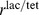 for the looped (λnull, red) and unlooped (λΔ*O_L_*, blue) controls. The PDF is estimated for 10-nm bins as described in the main text. Light-colored areas indicate 1 s.e.m. calculated by bootstrapping. (b) Cumulative density (CDF) of 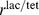 for the looped (λnull) and unlooped (λΔ*O_L_*) controls. The CDF is estimated for 10-nm bins as described in the main text. Light-colored area indicates 1 s.e.m. calculated by bootstrapping. (c) The PDF is shown for strains λWT (green), λ*O_R_*3^−^ (orange), and λ*O_L_*3^−^ (purple), calculated as in (a), and PDFs for strains λnull and λΔ*O_L_* are shown as dashed lines for comparison. (d) CDF estimates for three strains (dots; λWT, green; λ*O_R_*3^−^, orange; λ*O_L_*3^−^, purple) were fit as linear combinations of the positive (λnull) and negative (λΔ*O_L_*) control CDFs to estimate looping frequency. Colored lines indicate CDF fits and CDFs for strains λnull and λΔ*O_L_* are shown as dashed lines for comparison.

Visual inspection of the apparent separation between the LacI-mCherry and TetR-EYFP spots suggested that comparing the end-to-end separation in *O_R_*–*O_L_* DNAs required a more quantitative approach. We calculated 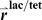 for all *O_R_*–O*_L_* DNA molecules in the λΔ*O_L_* and λnull strains that exhibited fluorescent spots in both EYFP and mCherry images. Figure 2f–h shows 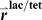 calculations for movies in Figure 2c–e, respectively, and Figure S3 shows 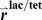 vectors for all movies lasting 0.8 s or longer. We then compiled the corresponding probability density distributions (PDF, 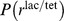, Figure 3a) and cumulative density distributions (CDF, 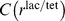, Figure 3b) of the vector magnitude, 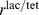. The long-tailed PDF observed for λnull (Figure 3a) is consistent with the expected end-to-end distance distribution measured from two spots with a fixed separation when the localization of each spot is subject to Gaussian fitting error [56]. A simple numerical simulation of the end-to-end distance PDF for two sites separated by 22 nm and each subject to 22-nm localization error largely recapitulates the long-tailed distribution (Figure S2c).

We found that the 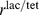 distribution for λΔ*O_L_* was distinctly different from that of λnull (*p*<10^−3^); the difference was reproduced in three independent experiments (Figure S1b). The mean separations, 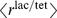, were 47 (*N* = 1,153) and 71 nm (*N* = 979) for λnull and λΔ*O_L_* respectively (results and measurement errors summarized in Table 2). Peaks in 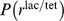 plots centered at ∼40 nm, reflecting our experimental precision in determining 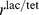; that is, *O_R_*–O*_L_* molecules with 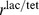 below 40 nm could not be distinguished from each other. Hence, it was more meaningful to compare distributions of 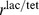 at large values where 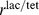 distributions differed most prominently. The cumulative probability of 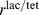 being 75 nm or more was ∼40% for λΔ*OL* and only ∼15% for λnull (Figure 3b). Furthermore, two-dimensional distributions of 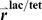 vectors (Figure S4) were clearly wider for λΔ*O_L_* than for λnull. Thus, by examining 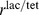 distributions, we could distinguish between the looped and unlooped control strains, suggesting that this approach could be used to probe CI-mediated DNA looping.

**Table 2:** Summary of measurements and sample statistics in this study.

Strain	*r* ^lac/tet^ Measurements	Mean *r* ^lac/tet^ (nm)	Median *r* ^lac/tet^ (nm)	Looping Frequency	CI Expression Level (WLU)
λnull	1,153	47±1	41±1	N/A	N/A
λΔ*O_L_*	979	71±1	63±2	N/A	1.38±0.05
λWT	962	52±1	45±1	79±6%	1.00±0.05
λ*O_R_*3^−^	784	59±1	50±2	53±7%	2.50±0.07
λ*O_L_*3^−^	781	56±1	48±1	60±7%	2.51±0.07

Errors are all 1 s.e.m. as estimated from 1,000 bootstrapped samples.

### Compact Conformation of Unlooped DNA λΔ*O_L_* Does Not Depend on Transcription or Nonspecific CI Binding

We measured the mean end-to-end separation 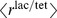 for λΔ*O_L_* at 71-nm, much shorter than the ∼200-nm distance expected for B-form DNA with a typical 50-nm in vitro persistence length [57]. While such a result is expected given the many factors known to compact prokaryotic chromosomes [58], it is possible that nonspecifically bound CI on the λΔ*O_L_* DNA and/or *P_RM_* transcription activity could influence the 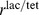 distribution, as indicated by a series of recent studies in vitro and in higher eukaryotic systems [46],[59],[60].

To examine these possibilities, we first compared the 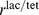 distribution of the λΔ*O_L_* strain to that of a control strain λΔ*O_L_P_RM_^−^cI^−^/cI^trans^* (Table 1, Figure S5a and b). In this control strain, promoter *P_RM_* was mutated to abolish transcription and the *cI* start codon was eliminated, but CI binding to *O_R_* was unaffected (Figure S5c, d, and e). In addition, we expressed CI from a plasmid at ∼9 times its level in λWT (Table S8). We found that the 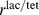 distributions of the λΔ*O_L_* and λΔ*O_L_P_RM_^−^cI^−^/cI^trans^* strains were indistinguishable (Figure S5a and b), demonstrating that the compact λΔ*O_L_* distribution does not depend on *P_RM_* transcription. Furthermore, 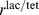 distributions for the same λΔ*O_L_P_RM_^−^cI^−^* strain with or without the CI-expressing plasmid were indistinguishable (Figure S5a and b), suggesting that nonspecifically bound CI did not interact with specifically bound CI at *O_R_* operator sites to condense DNA in vivo [46].

### In Vivo Observations of DNA Looping

We next investigated DNA looping in the context of wild-type and mutant *O_R_*–*O_L_* DNAs. In λWT, the wild-type λ sequence from *O_R_* through *O_L_* was inserted between *lacO^3^* and *tetO^3^*. CI could bind all *O_R_* and *O_L_* sites to mediate looping with both octameric and tetrameric CI complexes (Figure 1a). In λ*O_R_*3^−^ and λ*O_L_*3^−^, mutations in *O_R_*3 and *O_L_*3 essentially eliminated CI binding to these operators at lysogenic CI concentrations (Table 1) [35],[61].

We measured 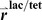 for these three strains and found that 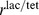 distributions differed significantly from those of the positive and negative controls λnull and λΔ*O_L_* (*p*<10^−3^, except *p* = 0.004 for λWT and λnull), with 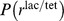 and 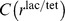 being intermediate to those of the controls (Figure 3c and d). Mean 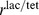 values for the three strains also fell in between those of λnull and λΔ*O_L_* (Table 2). The wild-type strain had lower 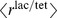 than λ*O_R_*3^−^ and λ*O_L_*3^−^, and its distribution differed from those of the mutant strains with moderate to high significance (*p* = 0.001 and 0.048 for λ*O_R_*3^−^ and λ*O_L_*3^−^, respectively); 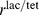 distributions for λ*O_R_*3^−^ and λ*O_L_*3^−^ were indistinguishable from each other (*p* = 0.493). The trend of λnull<λWT<λ*O_R_*3^−^≈λ*O_L_*3^−^<λΔ*O_L_* for 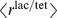 was reproduced in three independent experiments (Figure S1b). Assuming that a DNA molecule in the λWT, λ*O_R_*3^−^, and λ*O_L_*3^−^ strains is in either a looped or unlooped state, the intermediate 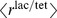 values of the three strains suggested that the fraction of looped DNA molecules (herein termed looping frequency) could be estimated by comparing 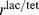 distributions of these strains to those of the looped and unlooped controls λnull and λΔ*O_L_*.

To further investigate whether the observed DNA looping in the λWT, λ*O_R_*3^−^, and λ*O_L_*3^−^ strains could be abolished by eliminating CI cooperative binding rather than by deleting *O_L_*, we constructed a control strain λCI^G147D^ (Table 1). This strain differs from λWT by a CI mutation G147D known to be defective in pairwise cooperative interaction [62],[63]. Structural evidence suggests that cooperative binding interfaces are shared for pairwise binding to adjacent operator sites and the formation of CI tetramers or octamers via DNA loops [64]. We found that the 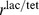 distribution of the λCI^G147D^ strain was indistinguishable from that of λΔ*O_L_* (Figure S5f and g, Table S7). We note that this G147D mutant also diminishes *P_RM_* transcription because of its weakened ability to form a CI tetramer at the O_R_1 and O_R_2 sites; hence its expression level is lower than that with wild-type CI (Table S8). Therefore, we constructed another control strain (λCI^G147D^/*cI^G147D,trans^*), in which the CI^G147D^ mutant protein was expressed constitutively at ∼11 times the CI expression level in λWT from a plasmid transformed into the λCI^G147D^ strain (Table S8). We found that 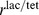 distribution of this strain was indistinguishable from that of the λΔ*O_L_* and the λCI^G147D^ strains, demonstrating that DNA looping could be abolished by eliminating CI cooperative binding.

### Estimating DNA Looping Frequency from 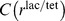


To quantitatively examine how operator mutations influence DNA looping, we estimated looping frequencies for λWT, λ*O_R_*3^−^, and λ*O_L_*3^−^ by assuming a simple model. In this model, DNA molecule can only exist in one of two states, looped or unlooped, with 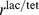 distributions for each state resembling those of the looped and unlooped controls, λnull and λΔ*O_L_*, respectively. Therefore, the distribution 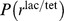 or 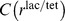 for one of the three strains is the linear combination of that of λnull and λΔ*O_L_*, with their distributions weighted by the looping frequency, 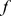:
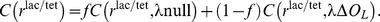
Using this model, we found that the looping frequency was 79% for λWT, and reduced to 53% for λ*O_R_*3^−^ and 60% for λ*O_L_*3^−^ (results with errors summarized in Table 2). The results were indistinguishable within error regardless of whether cumulative or probability density distributions were used, or whether data points from all frames or only the first frame of each molecule's movie were used (Table S1). The looping frequencies for λ*O_R_*3^−^ and λ*O_L_*3^−^ were indistinguishable from each other within error, suggesting a similar role of *O_R_*3 and *O_L_*3 in loop formation. Reduced looping frequencies of λ*O_R_*3^−^ and λ*O_L_*3^−^ compared to λWT suggest that while a CI octamer at *O_R_*12 and *O_L_*12 is sufficient to loop DNA, the resulting loop can be further stabilized by an additional CI tetramer only if both *O_R_*3 and *O_L_*3 are intact. To our knowledge, these measurements provide the first quantitative in vivo estimates of DNA looping frequencies that are independent of gene regulation models.

### Estimating DNA Looping Kinetics

In the above analyses, we only utilized 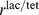, the magnitude of the 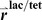 vector, and discarded information about the direction of 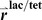 and its evolution in time. Looping frequencies estimated from 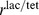 distributions are analogous to equilibrium constants and lack kinetic information. While many DNA molecules only exhibited fluorescent spots in both EYFP and mCherry channels for one or two consecutive frames due to photobleaching, some molecules had fluorescent spots lasting for several consecutive frames in both channels (Figure 2c–h; also see 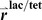 plots from molecules with many frames in Figure S3). By analyzing how 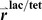 evolves in time, we can obtain additional information about DNA looping kinetics.

**Figure 4:**
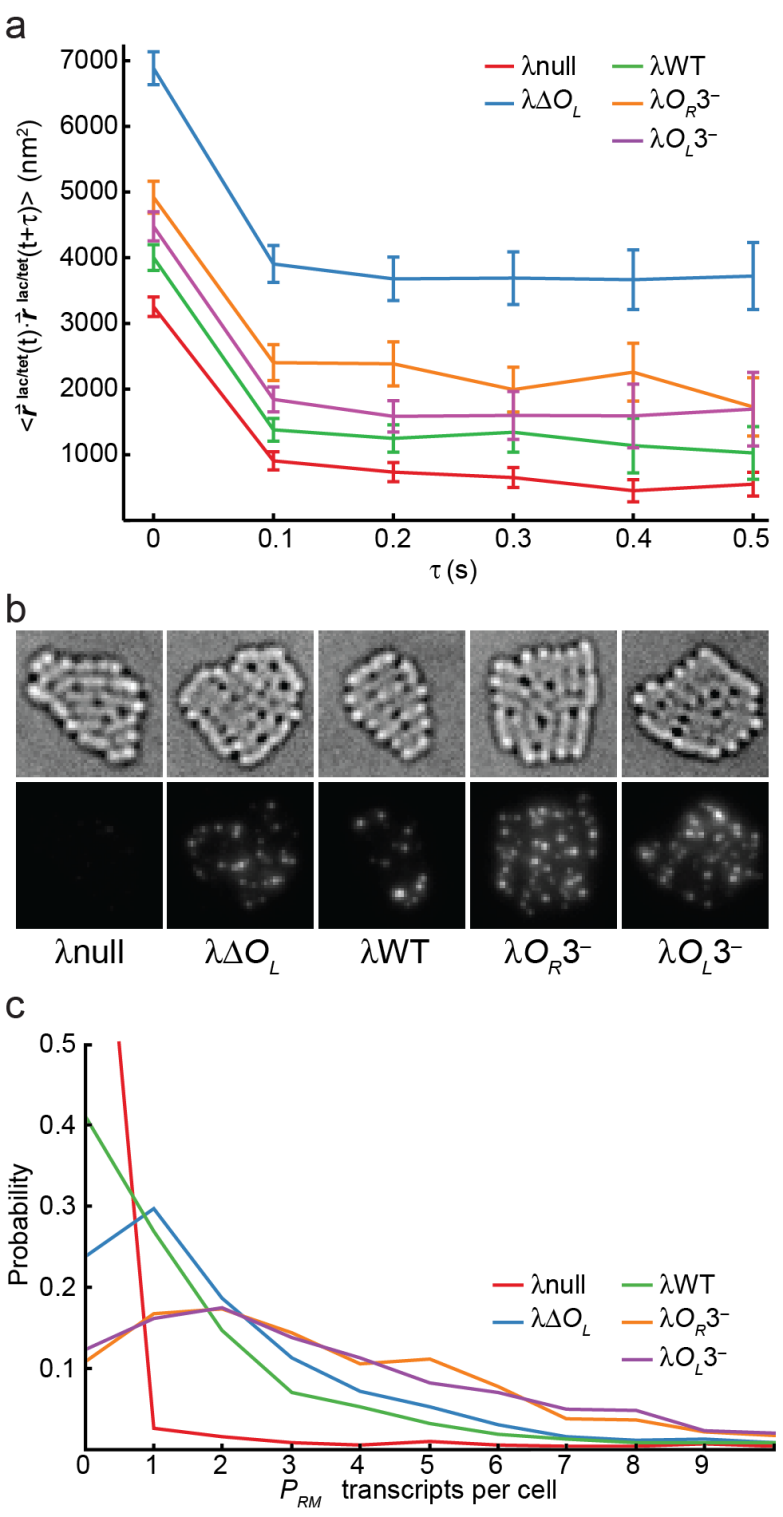
DNA looping kinetics and CI expression levels. (a) Vector dot-product autocorrelation for 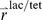 time trajectories for each strain. Plots show the average dot product of two 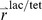 vectors separated by a given time lag. Error bars show 1 s.e.m. calculated by bootstrapping. (b) Typical smFISH images for λnull, which has no *P_RM_* transcripts, and for all other strains. Top, brightfield images showing a group of fixed cells for each strain. Bottom, maximum-intensity projections of fluorescence image stacks. Spots indicate one or more transcripts. (c) Distribution of *P_RM_* transcripts per cell determined by smFISH. The average expression level in wild-type λ units (WLU) is defined as the mean number of transcripts per cell in a given strain divided by the mean transcript number in λWT cells.

We calculated the autocorrelation of 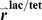 (the average dot product of two 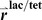 vectors separated by a time lag) up to 0.5 s for each strain using all movies in which fluorescent spots in both channels lasted two or more frames (Figure 4a). The autocorrelation curves of all strains showed an initial drop of ∼2,500 nm^2^ at the first time lag, corresponding to uncorrelated errors in determining 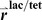. After the initial drops, all autocorrelation curves showed positive correlation values that were approximately constant at time lags up to 0.5 s.

The observation of near constant autocorrelation values after the first time lag for all the strains indicated that the conformation of each DNA molecule, characterized by both the magnitude and orientation of 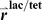, persisted for at least 0.5 s. This provides a lower limit for the amount of time it takes for two DNA sites in the relaxed, unlooped state to move relative to each other and potentially form a DNA loop, and thus an upper limit of ∼2 s^−1^ for the rate of DNA looping. The plateau values are related to the averaged mean end-to-end separations—λΔ*O_L_* has the highest autocorrelation plateau and λWT, λ*O_R_*3^−^, and λ*O_L_*3^−^ have intermediate values because they contain a mixture of looped and unlooped DNA molecules.

### Single-Molecule Measurements of CI Expression Levels

Next, we measured average CI expression levels, 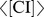, in all strains in order to understand to what different extent DNA looping influences *P_RM_* regulation. We used single-molecule fluorescence in situ hybridization (smFISH, [2],[65],[66]), in which multiple fluorescently labeled oligonucleotides probe targeted nonoverlapping regions of *cI* mRNA, to count the number of *P_RM_* transcripts in individual cells (Figure 4b and c). Given the assumption that the average number of CI molecules translated per *P_RM_* transcript is the same in all strains and the observation of indistinguishable cell growth rates (Figure S6a and b), we expected average mRNA expression levels proportional to 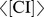. The λnull strain does not contain the *cI* gene and was used as a negative control. All other strains were transcriptionally active. Under our experimental conditions, the false positive rate using the λnull strain was ∼1 transcript per 50 cells, two orders of magnitude below the levels of all other strains; false positives arise when nonspecifically bound probes occasionally co-localize to create a fluorescent spot above the detection threshold. Typical smFISH images of the five strains are shown in Figure 4b. We quantified the number of transcripts in each individual cell by dividing the total intensity of fluorescent spots in each cell by the average intensity of a single-transcript spot (Figure 4c). We then determined 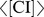 in wild-type λ units (WLU) by dividing the average number of transcripts in cells of a given strain by the average number of transcripts in λWT cells. We found that deleting *O_L_* increased 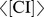 to ∼1.4 WLU (Table 2), indicating that the DNA loop formed between *O_L_* and *O_R_* in λWT enhances *P_RM_* repression. Mutating either *O_R_*3 or *O_L_*3 further increased 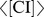 to ∼2.5 WLU. These observations are consistent with previous observations that although *O_L_*3 is 2.3 kb away from the *P_RM_* promoter, it has as important a role as *O_R_*3 in repressing *P_RM_* at lysogenic CI concentrations [13]. This suggests that *P_RM_* was not strongly repressed by CI binding to *O_R_*3 in the absence of a tetrameric interaction with an additional dimer at *O_L_*3. Finally, elevated 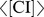 in λ*O_L_*3^−^ relative to λΔ*O_L_* indicated that DNA looping could also activate *P_RM_*, which was likely mediated by the binding of a CI octamer at *O_L_*12 and *O_R_*12, and was consistent with recent in vivo [49],[51] and in vitro [53] experiments.

### Evaluating Looping Free Energies and Transcription Activation Using a Thermodynamic Model

We have shown that reduced looping frequencies in λ*O_L_*3^−^ and λ*O_R_*3^−^ compared to that in λWT corresponded to increased expression levels of CI in the two strains, and that unlooped λΔ*O_L_* has a higher expression level than the λWT strain. To establish a quantitative framework that explains all observed relationships between looping and CI expression levels, we refined a thermodynamic model, with which we estimated looping free energies and the degree to which DNA looping changes the activity of *P_RM_*. These parameters are important because free energies describe the likelihood of interaction between two distal DNA sites, and changes in promoter activity directly reflect the influence of DNA looping on gene regulation.

The thermodynamic approach was first applied to model repression and activation of *P_RM_* by CI bound to *O_R_* [52] and recently modified to address looping [35],[44],[49],[51]. Our modeling approach is unique in that we used two independent, in vivo measurements, looping frequencies, and corresponding CI expression levels, to refine parameters for DNA-looping free energies and transcription activities. In previous modeling work, DNA-looping free energies were either inferred from *P_RM_* and *P_R_* expression-level measurements [35],[49],[51] or estimated using in vitro data [44].

The thermodynamic model and fixed physical parameters from previous reports we used to estimate *P_RM_* expression levels and DNA looping frequencies are essentially identical to the one used to analyze in vivo gene expression experiments [35]. Briefly, we assume that DNA states can be enumerated, that steady-state, in vitro DNA-binding measurements are applicable in vivo, and that mean expression rate, 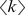, equals the sum of all products 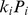, where 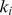 is the transcription rate in a particular state and 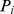 is the probability of the state at a given concentration of free CI dimers 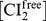:
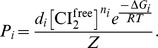
Each state is defined by its free energy, 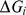, the number of bound CI dimers, 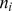, and the degeneracy, 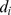, which is the number of states with the same 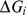, 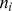, and 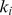. The model is described in greater detail in the Materials and Methods section; all states considered are listed in Table S2. 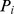 is normalized by the partition function, 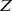, so that the sum of all state probabilities is 1. Following earlier work [49] and considering that the CI-mediated loop is relatively long, we assumed looping free energies to be independent of parallel or antiparallel orientation. Note that loop orientation is important in shorter DNA loops such as those mediated by Gal repressor [67]. We approximated the average CI concentration, 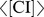, as the concentration at which the degradation rate equaled the production rate.

We refined our model to fit seven experimental observables: CI expression levels for λΔ*O_L_*, λWT, λ*O_R_*3^−^, and λ*O_L_*3^−^, and the looping frequencies for λWT, λ*O_R_*3^−^, and λ*O_L_*3^−^. We varied four free parameters: the free energies of forming a CI octamer and tetramer in the DNA loop as defined by Dodd et al. [35], 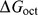, and 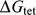, and the *P_RM_* expression rates when *O_R_*12 is bound by CI and DNA is either looped (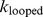) or unlooped (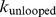). 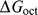 is the free energy of bringing together *O_R_* and *O_L_* when both are bound by two adjacent CI dimers to form a CI octamer, resulting in a looped conformation. 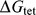 is the free energy of adding a CI tetramer to a loop already secured by a CI octamer. All other free energies and parameters such as specific and nonspecific DNA binding of CI were fixed at the values used by Dodd et al. [35]. The wild-type CI concentration was fixed to 220 nM (∼150 molecules/cell) based upon our previous experiment in which CI molecules were counted at the single-molecule level in a similar strain at similar growth conditions [3]. The CI degradation rate was fixed to give a half-life equal to the observed 2-h doubling time in our experiments.

**Figure 5:**
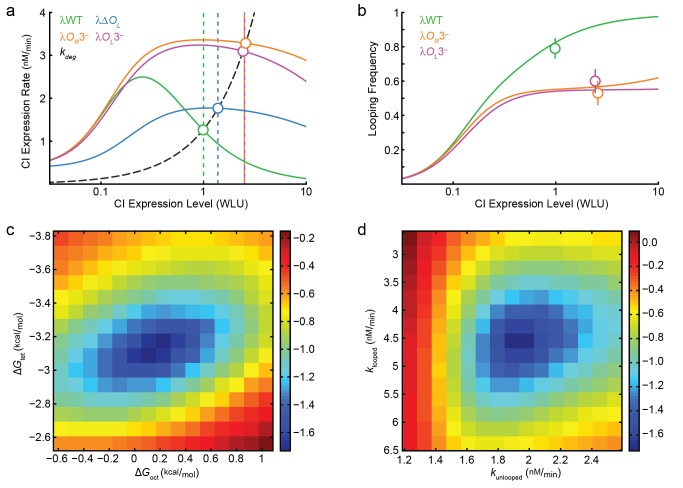
Thermodynamic modeling of *P_RM_* autoregulation by CI. (a) Measured and modeled CI expression rates as a function of CI expression levels in wild-type λ units (WLU; the concentration of CI molecules in the λWT strain). Colored curves indicate the modeled dependence of CI expression rates on CI expression levels and dashed black curve indicates the CI degradation rate. Modeled, steady-state CI expression levels are indicated by white circles where the degradation curve intersects CI-expression-level curves. Vertical dashed lines indicate measured CI expression levels (Table 2). (b) Measured and modeled looping frequencies as a function of CI expression levels. Curves show the dependence of looping frequencies on CI expression levels; white circles indicate modeled CI expression levels and measured looping frequencies for λWT, λO_R_3^−^, and λO_L_3^−^ with vertical lines indicating error in looping frequency estimates. (c, d) Fitting residual plots showing the uniqueness of best-fit model parameters. In each plot a parameter pair (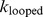 and 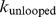 in c; 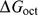 and 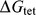 in d) is fixed, while the other parameter pair is varied and the corresponding minimum of the sum of squares of the difference between modeled and experimental parameters for all possible pairs was calculated. Parameter grids are colored according to the logarithm of the minimum sum of squares; well-defined minima indicate uniquely determined parameters.

The four free parameters were adjusted to best fit our experimental measurements of looping frequencies and CI expression levels. Modeled looping frequencies and CI expression rates at different CI concentrations are shown in Figure 5a and b. The best fit estimated 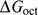 and 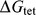 at 0.3 and −3.2 ^kcal^/_mol_, respectively, and the CI expression rates at 1.9 nM/s and 4.5 nM/s for unlooped (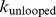) and looped (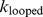) DNA when CI binds *O_R_*12. These results suggest that the DNA looping mediated by only a CI octamer is not strongly favored, while looping mediated by both an octamer and tetramer is the dominant configuration if all six binding sites are bound by CI dimers. Note that a small, positive 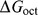 is consistent with measured looping frequencies greater than 50% for Δλ*O_L_*3^−^ and Δλ*O_R_*3^−^, as one unlooped configuration could lead to multiple looped configurations (Table S2). The higher CI expression rate from the looped configuration suggests that, in the absence of *O_R_*3 binding, bringing the distal *O_L_* and *O_R_* sites together to form a DNA loop activates *P_RM_* to 2.4 times the unlooped level.

To test how sensitive the fitting results were to two fixed parameters that are poorly defined in previous work, we varied CI expression levels and nonspecific DNA binding affinity. We found that across the examined ranges, octameric looping energies, 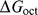, were consistently near 0 and tetrameric looping energies, 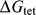, were strongly favorable between −2.8 to −4.6 ^kcal^/_mol_ (Table S3). Similarly, CI expression rates 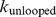 and 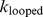 remained close to the original fit values, giving activation ratios between 1.7 and 2.5 (Table S3). We also verified that our fit parameters were unique—as shown in Figure 5c and d, the values of fit parameters corresponded to a well-defined minimum in the sum of squared residuals in the four-dimensional (two free energies and two expression rates) parameter space (Figure 5c and d). Hence we conclude that the four fit parameters resulted from the model were robust and well defined.

## Discussion

In this work, we directly measure the end-to-end separation between two DNA sites separated by only 2.3 kb on the *E. coli* chromosome with high spatial resolution, and report the first estimates of CI-mediated DNA looping frequencies in live *E. coli* cells. We improved a thermodynamic model to estimate the free energies of DNA looping as well as the degree to which DNA looping enhances transcription regulation. Combining independent, single-molecule measurements of looping frequencies and CI expression levels increased confidence in this model. Our results provide insight into transcription-factor-mediated DNA looping in vivo, and the new method reported here also has the potential to address questions beyond DNA looping, including understanding of chromosome structure and dynamics in vivo. In the following, we compare our results with previous work, and discuss unique information provided by our new method.

### Differences with in Vitro Looping Measurements

Our estimated looping frequencies of 79% for λWT and greater than 50% for λ*O_R_*3^−^ and λ*O_L_*3^−^ are larger than those observed in vitro by TPM and AFM, where looping frequencies at lysogenic CI concentrations were approximately 60% with wild-type operators and 10%–40% in the absence of *O_R_*3 and *O_L_*3 [42],[44],[46]. As looping frequency is directly linked to looping free energy, comparison of 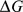 values showed the same trend: 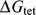 values estimated in these in vitro experiments were similar to our estimate of −3.2 ^kcal^/_mol_, while in vitro 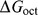 values were 1–2 ^kcal^/_mol_ higher than ours [44],[46].

Significantly different 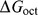 values likely resulted from differences between naked DNA in an in vitro environment and the compact, protein-decorated *E. coli* chromosome in the crowded cellular environment. Factors such as supercoiling and nonspecific, “histone-like” DNA-binding proteins could compact DNA and lead to more frequent encounters between *O_R_* and *O_L_*. Our observation that the unlooped λΔ*O_L_* DNA was extremely compact (discussed in more detail below) was consistent with this view; this level of compaction (comparable to a polymer with a 3-nm rather than a 50-nm persistence length) could lead to a 50-fold increase in the rate at which *O_R_* and *O_L_* encounter each other [68]. The relatively unchanged 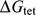 values could reflect the fact that the entropic and energetic costs of bringing *O_R_* and *O_L_* together are included in 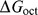. Our looping frequency estimates confirm what were predicted by in vivo gene expression experiments—DNA was estimated to loop ∼72% of the time for wild-type *O_R_*–*O_L_* DNA and ∼69% for DNAs similar to our λ*O_R_*3^−^ and λ*O_L_*3^−^ constructs [35]. Correspondingly, the 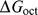 and 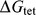 estimated in the in vivo work (−0.5 and −3.0 ^kcal^/_mol_) [35] compared well to ours (0.3 and −3.2 ^kcal^/_mol_).

One important assumption we employed in calculating looping frequencies is that that looped and unlooped λWT, λ*O_R_*3^−^, and λ*O_L_*3^−^ DNA molecules had similar 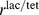 distributions to those of the looped control λnull and unlooped control λΔ*O_L_*, respectively. It is possible that the unlooped states in the λWT, λ*O_R_*3^−^, and λ*O_L_*3^−^ strains were more compact than that in λΔ*O_L_* if after a DNA loop breaks *O_R_*–*O_L_* DNA does not always completely relax before it reforms again. In such a case, looping frequencies estimated using the linear-combination model would be upper limits on the true looping frequencies. Nevertheless, as we show above, our looping frequency estimates broadly agree with expectations from previous studies. Since this simple model only requires one free parameter and gives reasonable results, it is unnecessary to invoke more complicated models.

### Effects of DNA Looping on Transcription Regulation

By comparing looping frequencies and corresponding CI expression levels in λWT, λΔ*O_L_*, λ*O_R_*3^−^, and λ*O_L_*3^−^, we showed that loop stabilization by the CI tetramer between *O_R_*3 and *O_L_*3 is important for efficient *P_RM_* repression, and that looping mediated by a CI octamer at *O_R_*1 and *O_R_*2 is important for *P_RM_* activation. We note that while it is possible that the presence of *tetO^3^* and *lacO^3^* binding sites flanking *O_R_*–*O_L_* DNA may influence CI binding and/or transcription, this influence is negligible. This is because CI expression levels in these strains measured using smFISH are comparable to that of a wild-type λ lysogen (Table S8), and our results are consistent with previous observations [13],[49],[51],[53]. Furthermore, results are directly comparable as all strains used in this study are identical with respect to the presence and positioning of these binding sites.

Combining these results in our thermodynamic model, we estimated that CI-mediated DNA looping activates *P*_RM_ to 2.4 times its level when the DNA does not loop. This compares well to earlier estimates of 2–4 fold [49], and 1.6-fold for a high-expression *P_RM_* mutant [53]. Another study did not find looping activates transcription, modeling CI-concentration-dependent *P_R_* and *P_RM_* activities without invoking activation via looping (by assuming 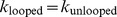) [35]. A later study indicated that this discrepancy may have resulted from different constructs used in the earlier study [49].

The molecular basis for DNA loop-enhanced *P_RM_* activation is unclear. One possibility is that a CI dimer bound to *O_R_*2 interacts with RNA polymerase to a greater extent if it is part of a higher-order CI octamer [53]. Alternatively, a recent work showed that a DNA UP element proximal to *O_L_* [49],[69] enhances CI expression from *P_RM_* in looped DNA by contacting the *α*-C-terminal domain of RNA polymerase [51]. The activation mechanism could be clarified in future experiments measuring both looping frequency and *P_RM_* activity while varying operator and UP element sequences and introducing CI mutations affecting operator binding, oligomerization, and RNA polymerase interaction.

### Kinetics of DNA Looping

We estimated the time scale a DNA molecule stays in a particular state by calculating the autocorrelation function of the 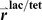 vector (Figure 4a). The 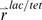 vector was strongly correlated for at least 0.5 s, suggesting that a particular DNA conformational state, either compact or extended, persisted for at least 0.5 s. This implies an upper limit of 2 s^−1^ for the rate of loop formation from the extended state. This upper bound of transition rate is in the range of what was observed in a previous TPM experiment, in which looped and unlooped states lasted for tens of seconds [44], and argues against a significantly faster rate used in a recent computer simulation (∼60 s^−1^) [50]. We note that although it is possible that transient CI unbinding does not necessarily lead to immediate and complete DNA conformational relaxation at our measurement time scale, the autocorrelation analysis puts an upper limit for the true transition rate between the looped and unlooped states. The same concern also applies to in vivo 3C and in vitro TPM experiments.

Slow transitions between looped and unlooped states imply that low or high expression states resulting from a particular DNA conformation could be long-lived, potentially committing a cell to a particular fate. Supporting this is a recent study that suggested that a single unlooping event could trigger induction of the *lac* operon [5]. We were unable to obtain time trajectories long enough to clearly identify looped/unlooped transitions for single DNA molecules. Development of brighter, faster maturing, and more photostable fluorescent proteins or in vivo labeling with synthetic fluorophores [70],[71] will help in increasing the number of measurements made on one DNA molecule, possibly enabling accurate measurement of DNA looping kinetics in vivo.

### The Short End-to-End Separation of λΔ*O_L_* Reflects the High Compactness of Chromosomal DNA

We observed very small end-to-end separation for the unlooped control (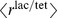 = 71 nm). This distance was shorter than expected from modeling the unlooped DNA as a noninteracting worm-chain with an in vitro persistence length of 50 nm [72], but consistent with the recently observed extreme bendability of short DNA molecules [73]. A noninteracting chain with an equivalent 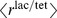 to that of λΔ*O_L_* would have a persistence length of only 3 nm, which is physically infeasible. Our measurements of indistinguishable conformational distributions in the absence of *P_RM_* transcription and the presence of CI overexpression suggest that neither transcription nor nonspecifically bound CI played a major DNA-compacting role in our experiments. Furthermore, *C. crescentus* chromosomal DNA segments of ∼5 kb were found to be similarly compact and consistent with Brownian dynamics simulations of supercoiled DNA [74].

We attribute the small end-to-end separation observed for λΔ*O_L_* to the high compaction of the *E. coli* chromosome in the crowded cellular environment. While the exact molecular mechanisms responsible for compaction remain unclear, previous studies found that in vitro binding of the histone-like HU proteins [75] (accession numbers P0ACF0, P0ACF4) and in vivo mammalian chromatin packing [76] reduced the apparent persistence length of DNA. Hence, it is possible that nucleoid-associated proteins such as HU may bring distal DNA sites together by protein–protein interactions and/or affect local DNA conformations by introducing bends and relieving torsional strain [77]. Another important factor could be negative supercoiling, which has been shown to compact the chromosomal DNA globally [78]. However, the exact effect of negative supercoiling on a 2.3-kb DNA segment is difficult to predict, because negative supercoiling could also introduce extended, plectonemic structures that promote large separations between DNA sites on relatively short length scales [78].

### Potential Applications

Our two-color, high-resolution method can be applied to examine how chromosomal location, DNA length, genetic background, and growth conditions affect the distance between any two DNA sites on the *E. coli* chromosome. Furthermore, the spatial organization of the *E. coli* chromosome can be determined by systematically measuring 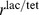 distributions between DNA sites throughout the chromosome. This method is similar to how chromosome conformation capture was used to generate a 3D model of the *C. crescentus* chromosome [79], but with significantly improved spatial resolution and without potential artifacts from fixation.

## Materials and Methods

### Strain and Plasmid Construction

A plasmid, pS2391, containing *lacO^3^* and *tetO^3^* (the *tetO_2_* sequence [54] was used for each repeat in *tetO^3^*) sites was synthesized by Genewiz, Inc. Segments of λ DNA (*O_R_* through *O_L_* for λWT, *O_R_* up to but not including *O_L_* for λΔ*O_L_*) from the wild-type lysogen JL5392 (a gift from John Little, University of Arizona) were amplified by PCR. This DNA was sequenced and inserted between *lacO^3^* and *tetO^3^* using the In-Fusion PCR cloning system (Clontech). A kanamycin-resistance cassette flanked by BamHI sites was amplified by PCR and inserted after *lacO^3^*. For strains with mutated operators, mutations r1 [80], *O_L_*3–4 [13], and *cI* ^G147D^ [62] were introduced to the λWT template via QuikChange (Agilent). A plasmid carrying the *P_RM_^−^cI^−^* mutations (Figure S5c) (λΔ*O_L_P_RM_* ^−^ *cI* ^−^) was constructed by overlapping PCR mutagenesis using complementary primers carrying the desired mutations, flanked by a forward primer that sits at the EcoRI site on the upstream end of the operon and a reverse primer at the ClaI site in the *rexA* gene downstream of *cI*. The 1.13 kb PCR product was introduced to the λΔ*O_L_* plasmid by restriction ligation.

This procedure resulted in seven plasmids that were used as templates in subsequent chromosome insertion: pZH105 (λnull), pZH016 (λΔ*O_L_*), pZH107 (λWT), pZH107r1 (λ*O_R_*3^−^), pZH107*O_L_*3–4 (λ*O_L_*3^−^), pACL006 (λWT^G147D^), and pACL007 (λΔ*O_L_P_RM_* ^−^ *cI* ^−^). Note that we use shorthand names such as λnull here for clarity; corresponding names used in our laboratory are listed in Table S4. The DNA sequence including *lacO^3^*, the λ DNA segment, *tetO^3^*, and the kanamycin resistance cassette was inserted into the chromosome of *E. coli* strain MG1655 by λ Red recombination [81], excising the *lac* operon, *lacI*, and all *lacO* sites.

To express the CI protein in trans from a plasmid, we constructed the plasmid pACL18 in which the wild-type *cI* ORF is driven by a constitutive promoter, *P_RM_^c^*, which has the wild-type −35 (TAGATA) and −10 (TAGATT) sequences, lacks *O_R_*2, and has a mutated *O_R_*1 sequence (CGCCTCGTGAGACCA) that eliminates binding by CI. The *pRM^c^–cI* fragment was then cloned to the ClaI site of the low-copy vector pACYC184. The plasmid pACL17 was generated similarly using a template containing the CI^G147D^ mutation.

The two-color reporter plasmid pLau53, which expresses LacI-ECFP and TetR-EYFP polycistronically under the control of the *P_BAD_* promoter [82], was obtained from the Yale Coli Genetic Stock Center. Because the autofluorescence spectrum of live cells is generally strongest at wavelengths around 500 nm [83], single-molecule imaging of blue-shifted fluorescent proteins such as ECFP is difficult. The red fluorescent protein mCherry, which further benefits from a large Stokes shift, fast chromophore maturation rate, and high brightness relative to other monomeric RFPs [84], was inserted in place of ECFP. We also created a tandem LacI-mCherry-EYFP reporter, which was used as a fiducial marker, by inserting the linker sequence from the tandem-dimer fluorescent protein tdTomato [84] in between mCherry and EYFP.

To accurately localize a fluorescent spot arising from only a few fluorescent protein molecules above the background of unbound molecules within a cell, we reduced the reporter expression level by weakening the ribosome binding sites (RBSs). Weakened RBS sequences were designed using an online RBS calculator [85]. For example, the RBS for TetR-EYFP translation was the consensus AGGAGG Shine-Delgarno sequence in the parent plasmid pLau53. Our reporter plasmid had an ACCAGG Shine-Delgarno sequence, with a predicted ∼300-fold decrease in the TetR-EYFP translation rate. All sequences including chromosome insertions were verified by sequencing (Genewiz Inc). Reporter plasmids are described in Table 1.

### Growth Condition

For all experiments reported in this study, cells were grown and imaged at room temperature (∼25°C) in M9 minimal media supplemented with MEM amino acids (Sigma). Cells were grown overnight with 0.4% glucose and 50 µg/ml carbenicillin to an optical density (OD_600_) of 0.4. After centrifugation at room temperature, cells were resuspended at OD600≈0.2 with 0.4% glycerol plus 0.2% L-arabinose and grown for 2 h (∼1 cell cycle) to induce LacI-mCherry and TetR-EYFP expression. Cells were again resuspended at OD_600_≈0.2 with 0.4% glucose and grown for another 2 h before immediate observation to allow time for fluorescent protein chromophores to mature.

We compared growth rates for the parent strain MG1655 to the experimental strain λnull to determine whether inserting the *lacO^3^* and *tetO^3^* construct into the chromosome and/or inducing expression from the reporter plasmid introduced a significant growth defect. Under induction growth conditions (∼27°C, M9 media with 0.4% glycerol and 1× MEM amino acids) starting at OD_600_≈0.1 and observing 8 h of growth, we measured doubling times of 2.7 h for MG1655 and 3.4 and 3.3 h for λnull harboring the reporter plasmid (in the absence and presence of 0.3% L-arabinose, respectively), indicating that there is no large growth defect associated with the insertion of the tandem operator sites into the chromosome and/or the expression of TetR-EYFP and LacI-mCherry fluorescent fusion proteins (Figure S6c).

### Imaging Conditions

In each experiment, samples of all strains were placed on separate gel pads in the same growth chamber. Two sets of at least 30 movies were acquired for each strain, with the second set acquired in the reverse order to minimize any bias possibly introduced by observing some strains in a particular order. All images were acquired within less than one cell doubling time.

Cells were put on a gel pad made of 3% low-melting-temperature SeaPlaque agarose (Lonza) in M9 with glucose and imaged on an Olympus IX-81 inverted microscope with a 100× oil immersion objective (Olympus, PlanApo 100× NA 1.45) and additional 1.6× amplification. Images were split into red and yellow channels using an Optosplit II adaptor (Andor) and captured with an Ixon DU-895 (Andor) EM-CCD with a 13-µm pixel width using MetaMorph software (Molecular Devices). Laser illumination was provided at 514 nm by an argon ion laser (Coherent I-308), which also pumped a rhodamine dye laser (Coherent 599) tuned to ∼570 nm. A quarter-wave plate (Thorlabs) was used to circularly polarize excitation light. Emitted light was split by a long-pass filter, and the red and yellow images were filtered using HQ630/60 and ET540/30 bandpass filters (Chroma).

### Measuring and Analyzing 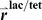


Images were inspected manually using a custom MATLAB script to identify spots that appeared in both EYFP and mCherry images. Images from all strains were displayed in random order without knowing the strain identify to avoid bias in spot selection. Pixel intensities within 3 pixels of the initial spot location were fitted with a symmetric, two-dimensional Gaussian distribution to estimate spot coordinates. The variance of the fit distribution was constrained to be less than 2 pixels. Spot-fitting error was estimated by scrambling residuals from a fit to the fluorescence data in 10 random permutations, adding them to the data, and fitting the resulting images; the reported error for a spot is the standard deviation of the distances between these fits and the initial fit to the raw data. Fitting error distributions are shown in Figure S1a.

The LacI-mCherry-EYFP tandem dimer (Figure S2b) in which the two fluorescent proteins were directly fused together was used to acquire fiducial control points to transform between the mCherry and EYFP coordinate systems. A projective transform was calculated from the control points using the cp2tform function in MATLAB. We found that relatively simple, global transformations were sufficient to transform coordinates of fluorescent beads (Tetraspeck, Invitrogen) with ∼10-nm registration error in our microscope setup, and did not see any further improvement with a locally weighted transformation used in in vitro two-color experiments [21]. This transformation was also used to generate the overlay images in Figure 2, Figure 3, and all supplemental movies. Fluorescent beads were not used as fiducial markers because the beads' emission spectra were different from those of the fluorescent proteins. Analysis was restricted to molecules in which mCherry and transformed EYFP coordinates were separated by less than 200 nm. Separations beyond this threshold were rare (∼1% of data, see two-dimensional distributions in Figure S4) and did not correlate with strain identity in any reasonable way. They possibly arose from data in which cells contained two labeled copies of *O_R_*–*O_L_* DNA.

After transformation into a uniform coordinate system, 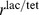 was calculated from the mCherry and EYFP coordinates and multiplied by an 81-nm pixel size (resulting from 160× magnification on a CCD with a 13-µm pixel width). Probability and cumulative distributions 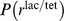 and 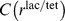 were calculated for 10-nm bins using the kernel smoothing probability density estimation (ksdensity) function in MATLAB, restricting the density to positive values and employing a uniform kernel width small enough to follow empirical cumulative density distributions without any systematic errors. Significant differences between 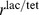 distributions were determined using a two-sample Kolmogorov–Smirnov test; two-tailed Student's *t* tests of sample means returned smaller, more significant *p* values. Errors in 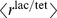 and 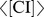 were determined by calculating the means of 1,000 bootstrapped samples; the reported error is the standard deviation of the calculated means. Looping frequencies were estimated by least squares fitting of 1,000 bootstrapped distributions (control distributions were also randomized on each iteration) and their error was calculated similarly.

### Single-Molecule Fluorescence in Situ Hybridization (smFISH)

Concentration measurements by smFISH followed a previously described protocol [66]. Transcripts from *P_RM_* were labeled with a mixture of 42 oligonucleotides labeled with CAL Fluor Red 610 (Biosearch Technologies), 31 of which hybridized to *cI* (11 targeted sequences not found in *E. coli* and did not cause a problematic level of false positives). Table S5 lists all 42 oligonucleotides. Labeled cells were imaged with 561-nm excitation at six imaging planes separated by 200 nm z-depth with negligible photobleaching. For each frame, fluorescent spots were automatically detected and fit to a Gaussian using a custom MATLAB routine. Nearly all molecules appeared in multiple image slices; the slice with the largest fit amplitude was kept. The integrated fluorescence of spots was observed to be quantized with one or a few molecules localized within one diffraction-limited spot. The intensity of one transcript was estimated from the distribution of spot intensities, and the number of molecules contributing to each spot was estimated from this quantization. The number of transcripts in each cell was estimated from the sum of the number of molecules in each spot within that cell. Alternatively, the number of molecules in one cell is proportional to its integrated fluorescence; this measurement provided the same average expression levels within error. The experiment was repeated to ensure that differences in labeling efficiency between samples were not responsible for differences in the number of detected molecules; combined data from both experiments were used for analysis.

### Simulation of 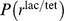


To generate simulated 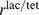 distributions, we first generated 10,000 random radial distances for a chain with a contour length 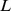 and persistence length 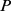 from a worm-like, noninteracting chain model using a Gaussian distribution with Daniels' approximation, which is accurate in the regime 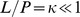 [72]:
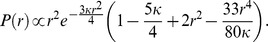
Each simulated 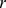 was projected onto the plane at a random angle to give a distance 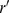. Simulated spots were placed at 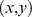 coordinates 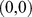 and 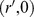. The MATLAB function mvnrnd was then used to simulate normally distributed measurement error with a standard deviation of 22 nm to the coordinates of each simulated spot. This procedure was sufficient to simulate the λnull distribution (Figure S2c) using a fixed end-to-end distance of 22-nm (approximate distance between the centers of the *lacO^3^* and *lacO^3^* sites; Figure S2a). Note here that the simulation is simplified in that it assumes that each spot has the same 22-nm localization error. In reality, localization error varies between different spots (Figure S1a) and there are other sources of measurement error. These differences may explain the slight deviation of the simulated distribution from the experimental distribution. The same procedure was used to estimate the 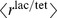 expected for 2.3-kb, B-form DNA with a 50-nm persistence (∼200 nm) as well as the apparently persistence length (3 nm) implied by the 71-nm 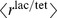 observed for λΔ*O_L_*.

### Thermodynamic Modeling

Additional descriptions of thermodynamic states are listed in Table S3. Parameter values were determined by first scoring a wide range of parameter values and iteratively searching narrower and more finely grained parameter ranges to manually minimize the sum of the squares of the differences between experimental and modeled values for looping frequency and CI expression level. We then refined this fit by least-squares minimization using MATLAB. This was done using a minimized model that only accounted for states likely to be populated near or above lysogenic CI concentrations (e.g., disregarding states in which *O_R_1* and *O_R_2* are unbound by CI). Using the same parameters and accounting for all 176 possible states (122 unique states accounting for degeneracy) did not significantly change the fit results. Fitting with this much more complex model gave octameric and tetrameric looping free energies of 0.6 and −3.3 ^kcal^/_mol_ and unlooped and looped expression rates of 2.1 and 5.3 nM/min. When determining parameters, rates were expressed in terms of changes in concentration per unit time; we followed earlier work in assuming that in a typical *E. coli* cell, a single molecule is at a concentration of ∼1.47 nM [35].

We do not report any estimate of fitting error; instead, we present only the parameters most consistent with our data and assumptions. Figure 5c and d shows that fit parameters were well-determined at a given combination of wild-type CI concentration and nonspecific binding parameters. As noted in the main text, varying these two parameters changed the absolute best-fit parameters, but did not dramatically change our conclusions. Furthermore, fixed parameters of previous studies were determined in a number of separate experiments employing different methods at temperatures other than 25°C; a rigorous estimate of modeling error would require knowing the error in the measurements of fixed parameters in our experimental conditions.

The basal CI expression rate, 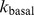, was arbitrarily fixed at 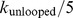; this did not have any significant impact on determining other parameters, as our measurements were all at or above lysogenic 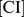, where *O_R_*2 is almost always bound by a CI dimer. Additionally, the fraction of free CI dimers was fixed at its value for 150 CI molecules per cell at a given concentration of nonspecific binding sites and nonspecific binding affinity. Fixing the concentration of free CI dimers is a reasonable approximation if (1) nearly all CI molecules are in dimers and (2) the number of free nonspecific binding sites is not significantly changed by nonspecifically bound CI dimers.

### Image Representation in Figures

Figure 2a–e, Figure 4b, and Movies S1, S2, S3, S4, S5, S6 were prepared using NIH ImageJ [86]. Raw fluorescence image intensities were scaled linearly from the lowest to highest values in region shown. For EYFP/mCherry overlay images, brightfield images were inverted and converted to 8-bit RGB. Fluorescence images were bandpass filtered and background subtracted before being used to generate magenta (mCherry) and green (EYFP) 8-bit RGB images that were added to the brightfield image. The EYFP images were first transformed in MATLAB using the imtransform function and the same fiducial data that were used to transform EYFP spot locations into mCherry coordinates. For smFISH images (Figure 4b), the value of each pixel is the maximum value of that pixel in six images collected at different *z*-axis positions. Intensities for all images were scaled linearly from the minimum to the maximum of all pictures (117–4,840 counts in 16-bit images).

## Supporting Information

Figure S1Spot fitting and experimental error analysis. (a) Distribution of fitting errors for EYFP (green), mCherry (red) localizations, and 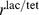 (black). Errors were estimated using a bootstrapping procedure by fitting raw data to a Gaussian distribution. The residuals from this fit were then randomly rearranged and added back to the data in 10 different permutations. The reported error is the standard deviation of the distance between these 10 locations and the initial fit location. Error in 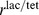 was determined similarly; from the 10 bootstrapped EYFP and mCherry fits, 100 distances were obtained and the error was estimated as the standard deviation of the difference between these distances and the distance determined from fitting the raw data. (b) A compilation of all data from three separate experiments was used for all analysis in the main text. Here, 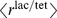 is shown for the individual experiments. Error was estimated as the standard deviation of the means of 1,000 bootstrapped distributions. Except for one sample (λ*O_R_*3^−^, day 3), the estimated mean separations for all days followed the trend 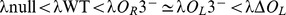.(TIF)

Figure S2Estimate of positive control dimensions and apparent end-to-end distance distribution. (a) The maximum distance between TetR-EYFP and mCherry-LacI chromophores was approximated assuming straight DNA. All distances are in nm. Here, bound fusion proteins are shown on the same face of a DNA molecule, but this needs not be the case. Dimers of DNA-binding proteins were based on Protein Data Bank (PDB) entries for TetR (1QPI [87]) and LacI (1EFA [88]). Both fluorescent proteins are shown using the entry for GFP (1GFL [89]). Protein structures images generated using VMD [90]. (b) In an alternative positive control that was used to collect fiducial data for image registration, the plasmid pZH102R33TD encodes the tandem-dimer reporter LacI-mCherry-EYFP. (c) The 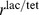 PDF for the λnull control (black line; 1 s.e.m. shown in red as in Figure 3a) is shown with the distribution of 10,000 numerically simulated end-to-end distances for two sites separated by 22 nm, randomly projected onto the 2D plane, and subjected to 22-nm localization error for both ends (dashed black line). PDFs were calculated using methods described in main text. See Materials and Methods for simulation details.(TIF)

Figure S3Plots showing trajectories of 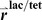 vectors for all data from all strains for every molecule that was fit in both the EYFP and mCherry images for at least 8 consecutive frames (800 ms). Green and magenta lines are single-color trajectories for TetR-EYFP and LacI-mCherry spots, respectively; the corresponding 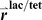 trajectory with time colored-coded from blue to red is plotted on top at the same length scale. Coordinates are in nm.(PDF)

Figure S4Two-dimensional distributions of the x and y components of 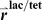 vectors. (a) A cartoon describes the calculation of the x and y components of the 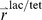 vector. In the projected image, the 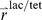 vector has two components determined by the arbitrary orientation of the detector. (b–f) Heat maps of the distribution of the x and y components of 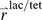 vectors of each strain. Plots were generated by binning the data for all 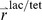 into 5 nm×5 nm bins. The resulting 2-dimensional distribution was then filtered with a Gaussian kernal (with a width similar to spot-localization precision) to approximate the smoothed distributions. Each image is colored by the probability of the 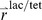 vector falling within a given bin according to the scale bar in (b).(TIF)

Figure S5Experiments showing the effects of transcription, nonspecific CI binding and higher-ordered CI oligomer on DNA looping. (a) End-to-end distance (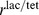) distributions (PDF) for λnull (red), λΔ*O_L_* (blue), λΔ*O_L_P_RM_^−^cI^−^* (purple), and λΔ*O_L_P_RM_^−^cI^−^/cI^trans^* (green). The PDF is estimated for 10-nm bins. (b) Cumulative density of 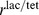 (CDF) for λnull (red), λΔ*O_L_* (blue), λΔ*O_L_P_RM_^−^cI^−^* (purple), and λΔ*O_L_P_RM_^−^cI^−^/cI^trans^* (green). The CDF is estimated for 10-nm bins. (c) DNA sequence for the *P_RM_^−^cI^−^* mutant in comparison to the wild-type sequence. Mutated nucleotides are shown in red. (d) Gel shift assay monitoring the binding of wild-type CI protein. Lane 1–4, CI at concentrations of 0, 150, 300, and 600 nM binding to a 158-bp DNA fragment (20 nM) amplified from the plasmid pZH107 carrying the wild-type P*_RM_* DNA sequence. Lane 5–8, CI at concentrations of 150, 0, 300, and 600 nM (note loading order) binding to a 158-bp DNA fragment (20 nM) amplified from the plasmid pACL007 carrying the *P_RM_^−^cI^−^* sequence. Lane 9: empty. Lane 10–13, CI at concentrations of 0, 150, 300, and 600 nM binding to a 140-bp DNA fragment (20 nM) amplified from the *E. coli hns* promoter region, which CI does not bind specifically. Reaction mixtures were incubated in a buffer (10 mM Tris pH 8.0, 50 mM KCI, 1 mM MgCl_2_, 10% glycerol, 100 ug/ml BSA, 1 mM DTT) at room temperature for 10 min. Samples were electrophoresed in Bio-Rad 4–20% Gradient TBE gels (Bio-Rad, Hercules, CA) in a cold room and then stained with Ethidium Bromide for 30 min. (e) Fraction of bound DNA (intensity of low-weight band divided by intensity of lane over background) quantified using NIH ImageJ for the gel shown in (d). (f, g) Distributions of 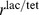 identical in description to those in (a, b) showing strains λnull (red), λΔ*O_L_* (blue), λG147D (purple), and λG147D/*cI^G147D,trans^* (green).(TIF)

Figure S6 Growth rate comparisons. (a, b) Strains used in thermodynamic modeling were diluted from exponential growth to low optical densities in M9 minimal media supplemented with 0.4% glucose and carbenicillin as described in the main text. OD600 was measured over 10 h of growth for two replicate experiments. Strains are λΔ*O_L_* (blue), λWT (red), λ*O_R_*3^−^ (green), and λ*O_L_*3^−^ (purple). Doubling times calculated using the Microsoft Excel LOGEST function range from 1.7 to 2.5 h. Two independent replicates are shown. (c) Growth rates for the parent *E. coli* strain MG1655 (blue) were compared to those of the control strain λnull in which the *lac* operon is replaced with a construct incorporating the *lacO^3^* and *tetO^3^* binding site arrays and which harbors the plasmid pZH102R33Y29 which expresses both TetR-EYFP and LacI-mCherry fluorescent fusion proteins upon arabinose induction. Strains were grown in M9 minimal media supplemented with 0.4% glycerol and λnull was grown in both the absence (red) and presence (green) of 0.3% L-arabinose. Doubling times were 2.7 h for MG1655 and 3.4 and 3.3 h for λnull in the absence and presence of L-arabinose, respectively.
(TIF)

Movie S1 Fluorescence movie montage for strain λnull corresponding to the data in Figure 2c. Single-color images for TetR-EYFP (top left) and LacI-mCherry (top right) data have intensities scaled linearly from the lowest to the highest pixel values in the first image in each time series. Before creating the overlay images (bottom), single-color images were background subtracted and bandpass filtered using the program ImageJ [91]. The overlay images are scaled to be twice as large as the single-color images. Scale bars correspond 4 µm in the small, single-color images and 2 µm in the overlay image. Ten consecutive image frames are shown in real time (10 frames per second); the movie is looped 5 times.
(MOV)

Movie S2 Fluorescence movie montage for strain λΔ*O_L_* corresponding to the data in Figure 2d. Single-color images for TetR-EYFP (top left) and LacI-mCherry (top right) data have intensities scaled linearly from the lowest to the highest pixel values in the first image in each time series. Before creating the overlay images (bottom), single-color images were background subtracted and bandpass filtered using the program ImageJ [91]. The overlay images are scaled to be twice as large as the single-color images. Scale bars correspond 4 µm in the small, single-color images and 2 µm in the overlay image. Ten consecutive image frames are shown in real time (10 frames per second); the movie is looped 5 times.
(MOV)

Movie S3 Fluorescence movie montage for strain λΔ*O_L_* corresponding to the data in Figure 2e. Single-color images for TetR-EYFP (top left) and LacI-mCherry (top right) data have intensities scaled linearly from the lowest to the highest pixel values in the first image in each time series. Before creating the overlay images (bottom), single-color images were background subtracted and bandpass filtered using the program ImageJ [91]. The overlay images are scaled to be twice as large as the single-color images. Scale bars correspond 4 µm in the small, single-color images and 2 µm in the overlay image. Thirteen consecutive image frames are shown in real time (10 frames per second); the movie is looped 5 times.
(MOV)

Movie S4 Fluorescence movie montage for strain λWT corresponding to a typical, long movie. Single-color images for TetR-EYFP (top left) and LacI-mCherry (top right) data have intensities scaled linearly from the lowest to the highest pixel values in the first image in each time series. Before creating the overlay images (bottom), single-color images were background subtracted and bandpass filtered using the program ImageJ [91]. The overlay images are scaled to be twice as large as the single-color images. Scale bars correspond 4 µm in the small, single-color images and 2 µm in the overlay image. Twelve consecutive image frames are shown in real time (10 frames per second); the movie is looped 5 times.
(MOV)

Movie S5 Fluorescence movie montage for strain λ*O_R_*3^−^ corresponding to a typical, long movie. Single-color images for TetR-EYFP (top left) and LacI-mCherry (top right) data have intensities scaled linearly from the lowest to the highest pixel values in the first image in each time series. Before creating the overlay images (bottom), single-color images were background subtracted and bandpass filtered using the program ImageJ [91]. The overlay images are scaled to be twice as large as the single-color images. Scale bars correspond 4 µm in the small, single-color images and 2 µm in the overlay image. Thirteen consecutive image frames are shown in real time (10 frames per second); the movie is looped 5 times.
(MOV)

Movie S6 Fluorescence movie montage for strain λ*O_L_*3^−^ corresponding to a typical, long movie. Single-color images for TetR-EYFP (top left) and LacI-mCherry (top right) data have intensities scaled linearly from the lowest to the highest pixel values in the first image in each time series. Before creating the overlay images (bottom), single-color images were background subtracted and bandpass filtered using the program ImageJ [91]. The overlay images are scaled to be twice as large as the single-color images. Scale bars correspond 4 µm in the small, single-color images and 2 µm in the overlay image. Twelve consecutive image frames are shown in real time (10 frames per second); the movie is looped 5 times.
(MOV)

Table S1 Looping frequencies were estimated from alternate data sets using either all data or only the data from the first frames (for molecules appearing in more than one sequential frame) and fitting either probability (PDF) or cumulative (CDF) distributions. The first row results for each strain were reported in the main text.
(DOCX)

Table S2States used in thermodynamic modeling. We used free-energy parameters that were described by Dodd et al. [35]. States that will not be populated near lysogenic CI concentrations (e.g., those without *O_L_*1 or *O_L_*2 bound) are ignored; the reference state (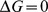) has CI dimers bound to *O_L_*1 and *O_L_*2. A state with *O_R_* free of CI is included to show activation in Figure 5a and b, but does not significantly change fit parameters; because *O_R_*1 and *O_R_*2 binding is highly cooperative, we do not model states with only one or the other operator bound. The degeneracy term indicates how many microstates exist with identical CI dimer binding patterns and free energies. A particular macrostate may have several microstates that differ in terms of parallel or antiparallel looping configurations or in the identity of binding sites participating in cooperative interactions (either through looping or through adjacent dimers). Here, we also list whether a state is looped (1 for looped; 2 for unlooped) as well as its transcription rate, 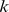 (0; 1 for 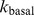; 2 for 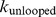; 3 for 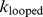). The free energy of state 2 is called 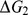 below.(DOCX)

Table S3 Thermodynamic model fitting using alternative choices for wild-type CI concentration (expressed here in molecules/cell; in the model, 1 molecule per cell is equivalent to 1.47 nM) and the fraction of CI molecules that are in the form of free dimers. The approximation of a constant free-dimer fraction is reasonable if specifically bound CI dimers (up to 6 dimers composed of 12 monomers) do not make up a large fraction of total CI and if CI concentration is sufficiently high that almost all CI molecules are in dimeric complexes. The free-dimer fractions used here were calculated assuming the absence of specific binding sites using the parameters for nonspecific binding site affinity and concentration estimated by Dodd et al. [35]. Results in the first row are the same as those presented in the main text.
(DOCX)

Table S4 Names of new strains used in this study (as used internally in our lab) and shorthand names used in the main text.
(DOCX)

Table S5 Sequences of oligonucleotide probes for single-molecule fluorescence in situ hybridization (smFISH) experiment. Asterisks indicate probes that do not hybridize specifically with any *E. coli* sequence. All other probes hybridize nonoverlapping sequence in the *cI* coding region of the mRNA transcript from the *P_RM_* promoter.
(DOCX)

Table S6Measurement statistics for experiment comparing 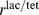 distributions for looped and unlooped control strains to 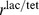 for strains lacking *O_L_* and having weakened *P_RM_* promoters with and without the overexpression of wild-type CI from a plasmid. Errors for the 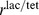 measurements are all 1 s.e.m. as estimated from 1,000 bootstrapped samples. Note that 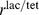 distributions display small, day-to-day variability between experiments (see Figure S1, this table, Table 2, Table S7), but the trend stays the same for a given set of experiments.(DOCX)

Table S7Measurement statistics for experiment comparing 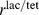 distributions for looped and unlooped control strains to 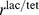 for strains in which CI harbors the G147D mutation with and without the overexpression of CI^G147D^ from a plasmid. Errors for the 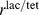 measurements are all 1 s.e.m. as estimated from 1,000 bootstrapped samples. Note that 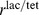 distributions display small, day-to-day variability between experiments (see Figure S1, this table, Table 2, Table S6), but the trends stays the same for a given set of experiments.(DOCX)

Table S8 CI expression levels measured by smFISH for wild-type phage lambda lysogen JL5392 and additional strains. For strains with replicate experiments (N, number of independent experiments), errors indicate standard deviation. The expression levels were normalized to wild-type units (WTUs) using the λWT strain.
(DOCX)

## Abbreviations

3C: chromosome conformation capture
CDF: cumulative distribution function
PDF: probability density function
RBS: ribosome binding site
smFISH: single-molecule fluorescence in situ hybridization
TPM: tethered partial motion
WLU: Wild-type λ units
