# Supplementary figures and images for: Transcription-Factor-Mediated DNA Looping Probed by High-Resolution, Single-Molecule Imaging in Live E. coli Cells
Source: PLoS Biol. 2013 Jun 18;11(6):e1001591. doi: 10.1371/journal.pbio.1001591 (PMC3708714; doi:10.1371/journal.pbio.1001591)

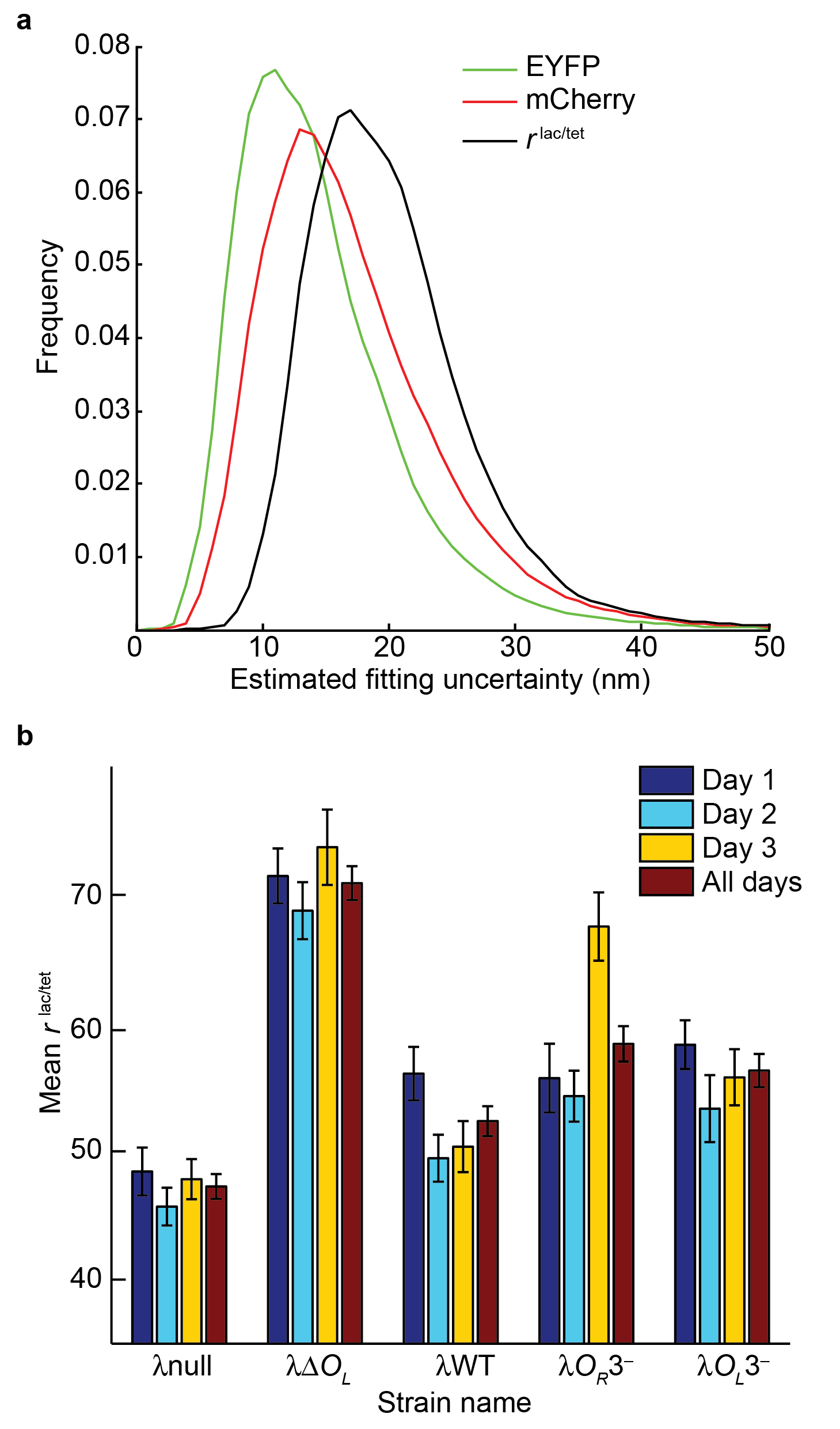

Supplement: Figure S1 — Spot fitting and experimental error analysis. (a) Distribution of fitting errors for EYFP (green), mCherry (red) localizations, and (black). Errors were estimated using a bootstrapping procedure by fitting raw data to a Gaussian distribution. The residuals from this fit were then randomly rearranged and added back to the data in 10 different permutations. The reported error is the standard deviation of the distance between these 10 locations and the initial fit location. Error in was determined similarly; from the 10 bootstrapped EYFP and mCherry fits, 100 distances were obtained and the error was estimated as the standard deviation of the difference between these distances and the distance determined from fitting the raw data. (b) A compilation of all data from three separate experiments was used for all analysis in the main text. Here, is shown for the individual experiments. Error was estimated as the standard deviation of the means of 1,000 bootstrapped distributions. Except for one sample (λOR3−, day 3), the estimated mean separations for all days followed the trend . (TIF) [file pbio.1001591.s001.tif]

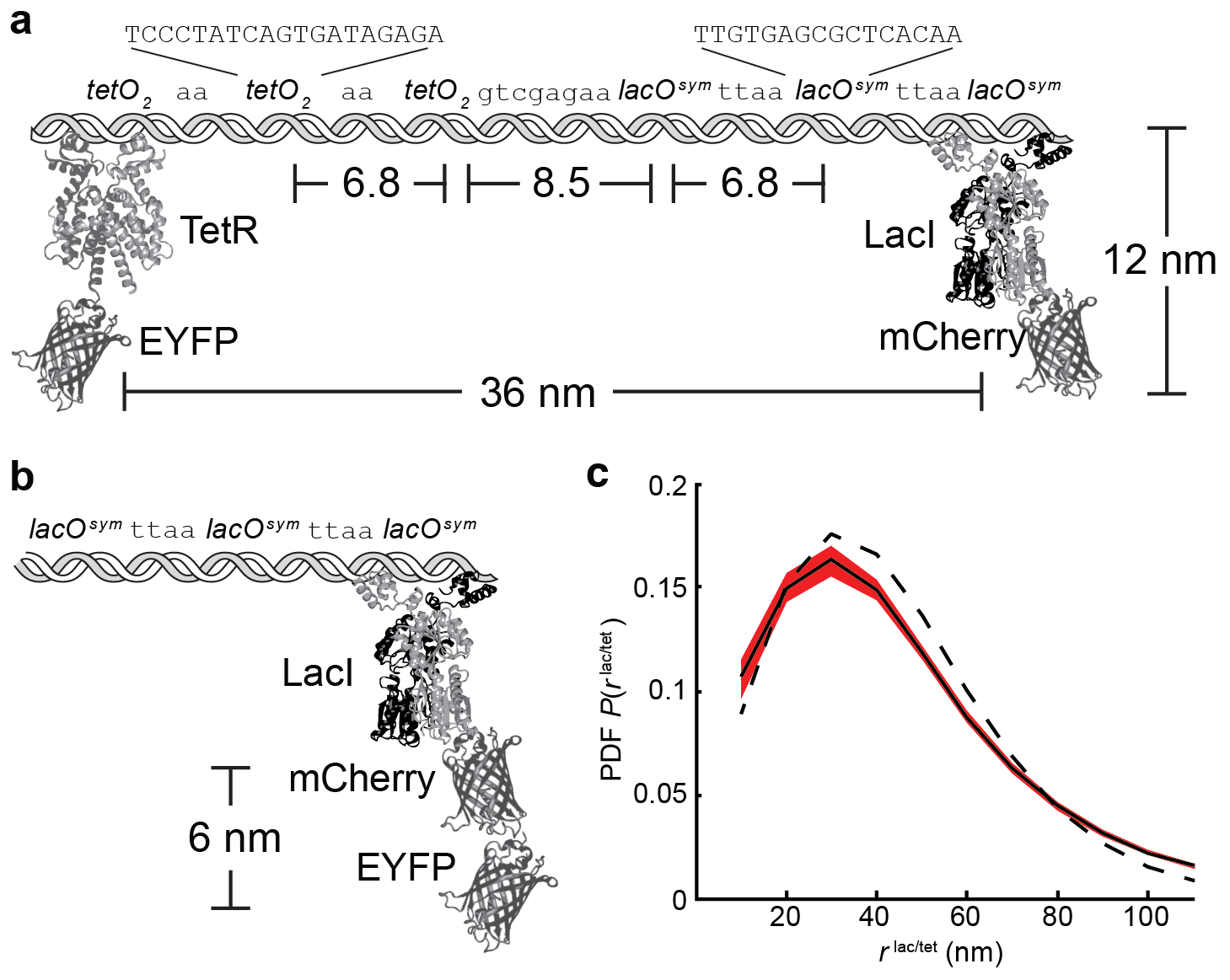

Supplement: Figure S2 — Estimate of positive control dimensions and apparent end-to-end distance distribution. (a) The maximum distance between TetR-EYFP and mCherry-LacI chromophores was approximated assuming straight DNA. All distances are in nm. Here, bound fusion proteins are shown on the same face of a DNA molecule, but this needs not be the case. Dimers of DNA-binding proteins were based on Protein Data Bank (PDB) entries for TetR (1QPI [87]) and LacI (1EFA [88]). Both fluorescent proteins are shown using the entry for GFP (1GFL [89]). Protein structures images generated using VMD [90]. (b) In an alternative positive control that was used to collect fiducial data for image registration, the plasmid pZH102R33TD encodes the tandem-dimer reporter LacI-mCherry-EYFP. (c) The PDF for the λnull control (black line; 1 s.e.m. shown in red as in Figure 3a) is shown with the distribution of 10,000 numerically simulated end-to-end distances for two sites separated by 22 nm, randomly projected onto the 2D plane, and subjected to 22-nm localization error for both ends (dashed black line). PDFs were calculated using methods described in main text. See Materials and Methods for simulation details. (TIF) [file pbio.1001591.s002.tif]

Figure S3

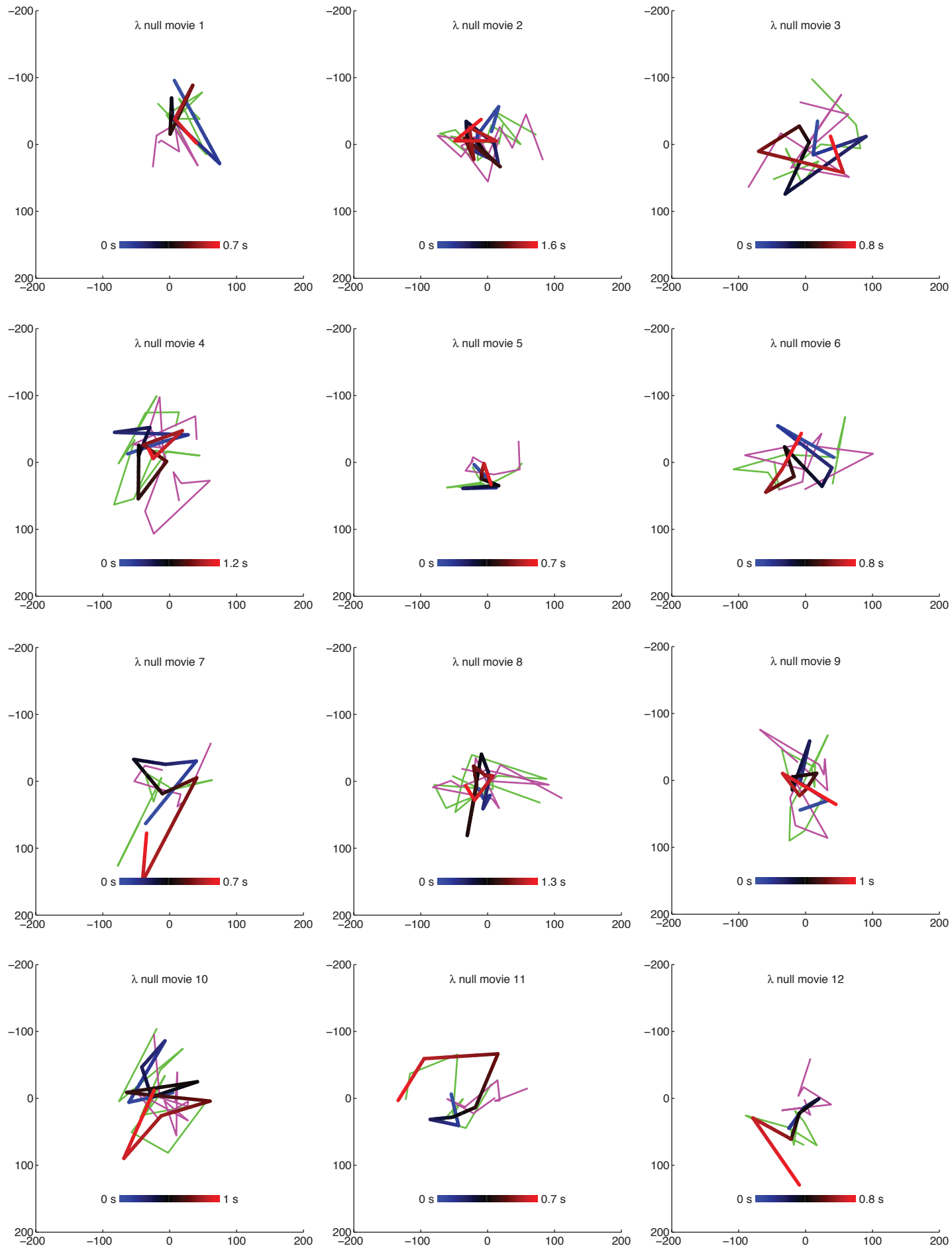

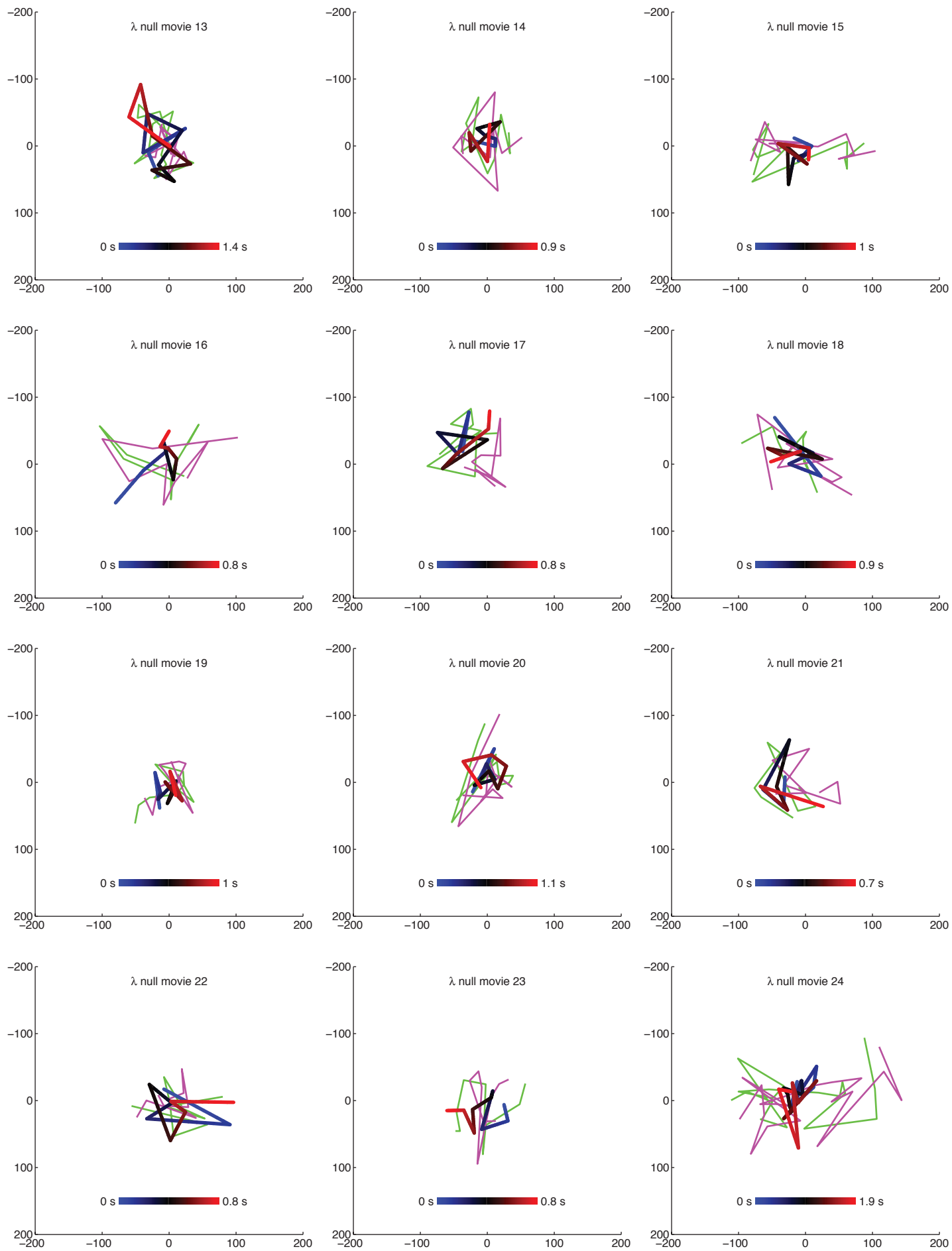

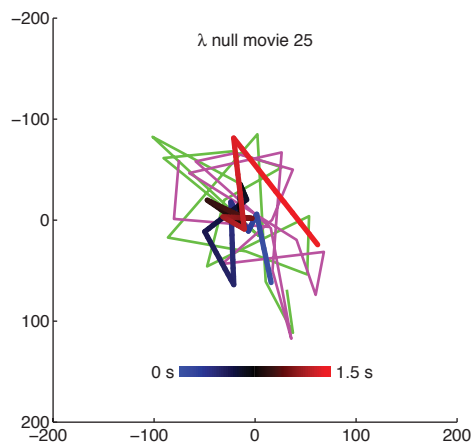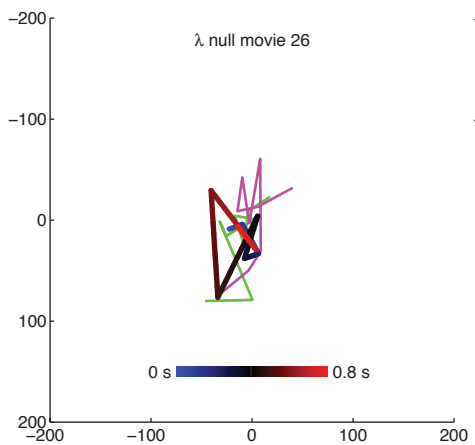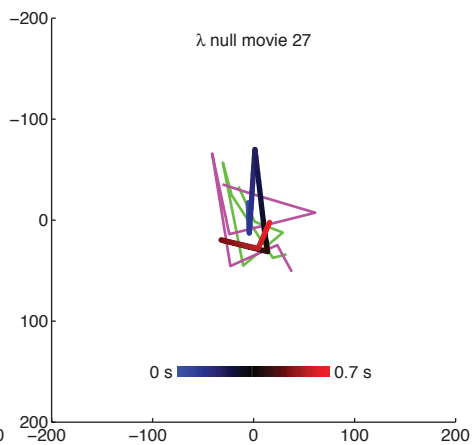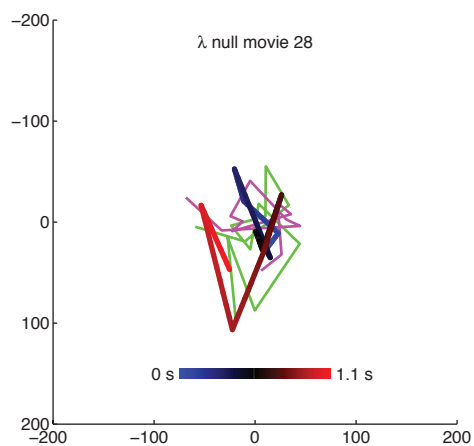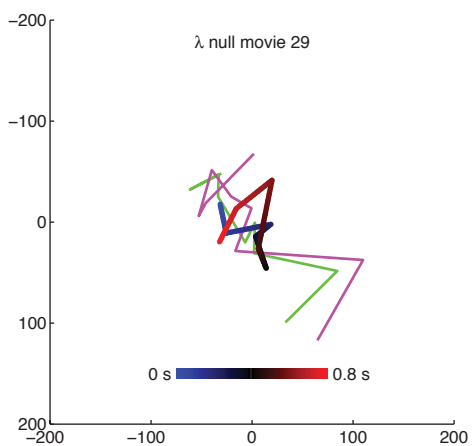

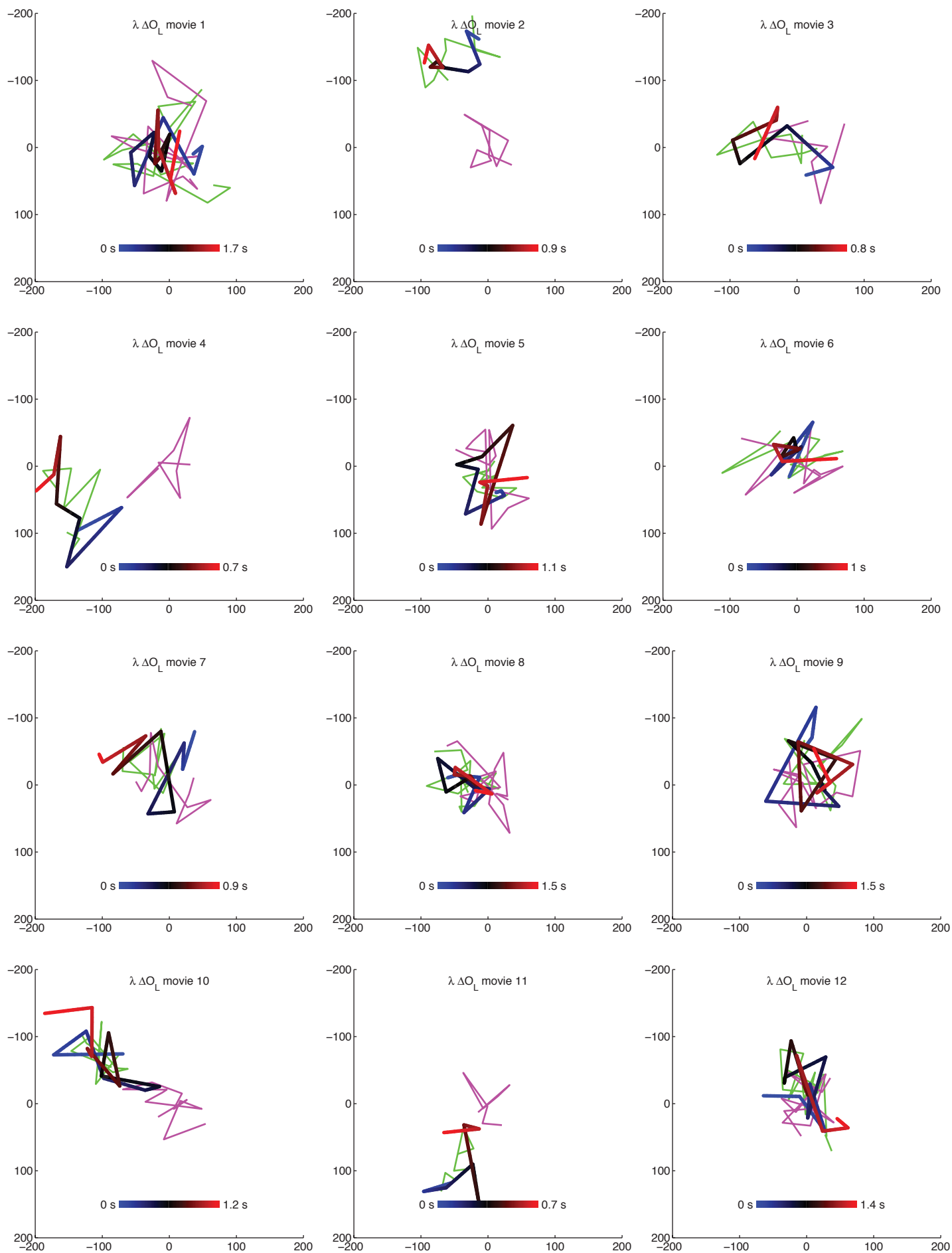

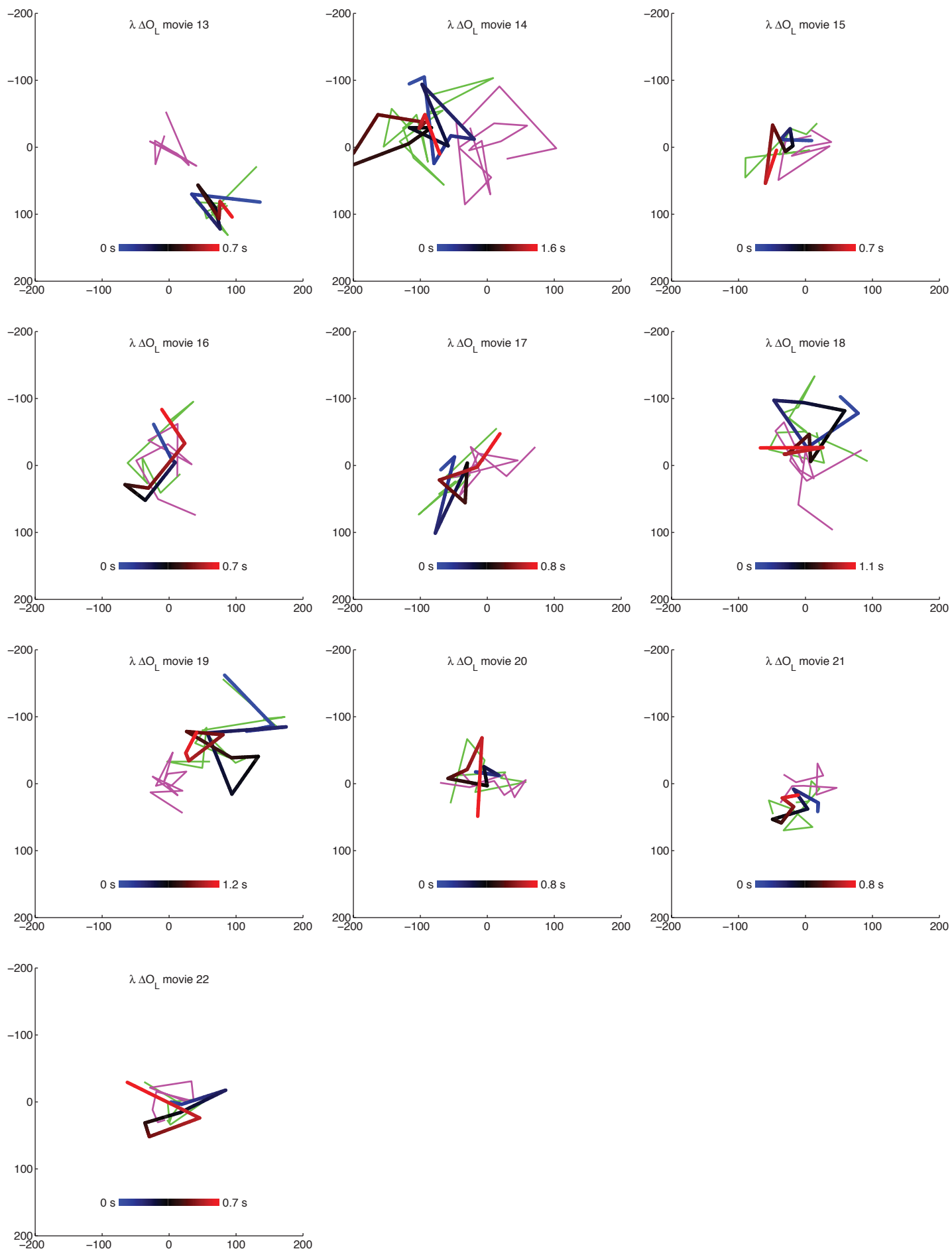

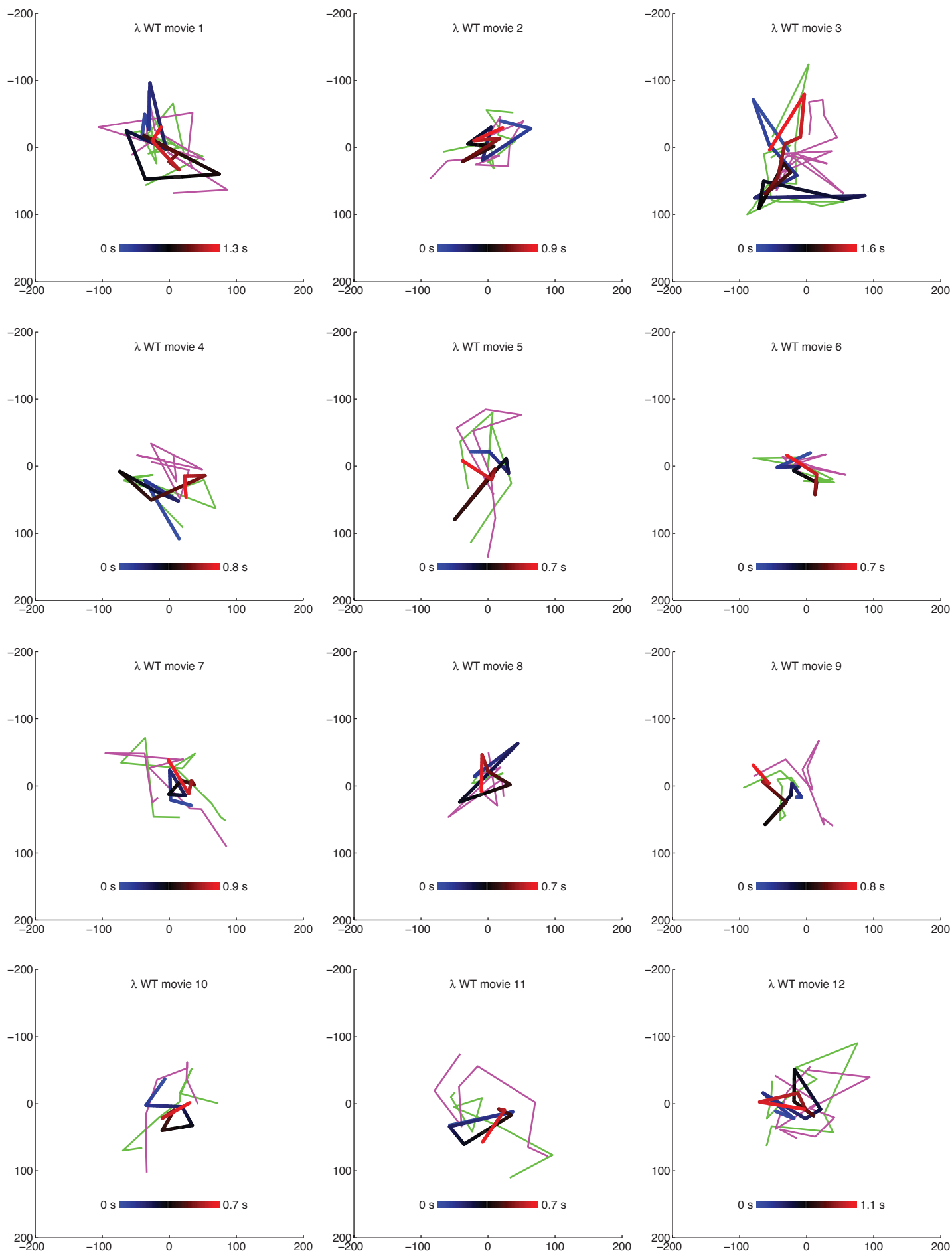

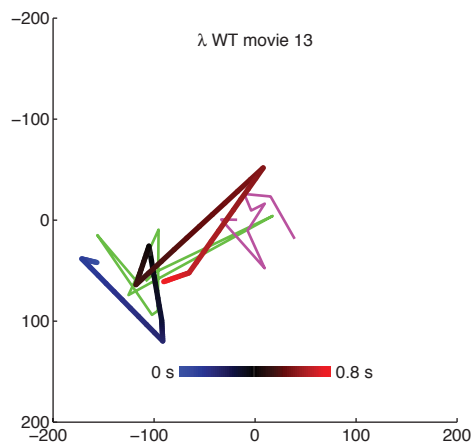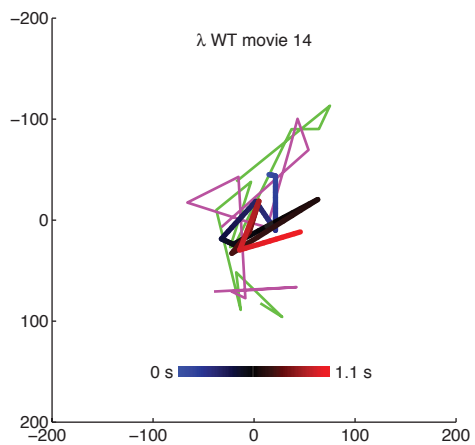

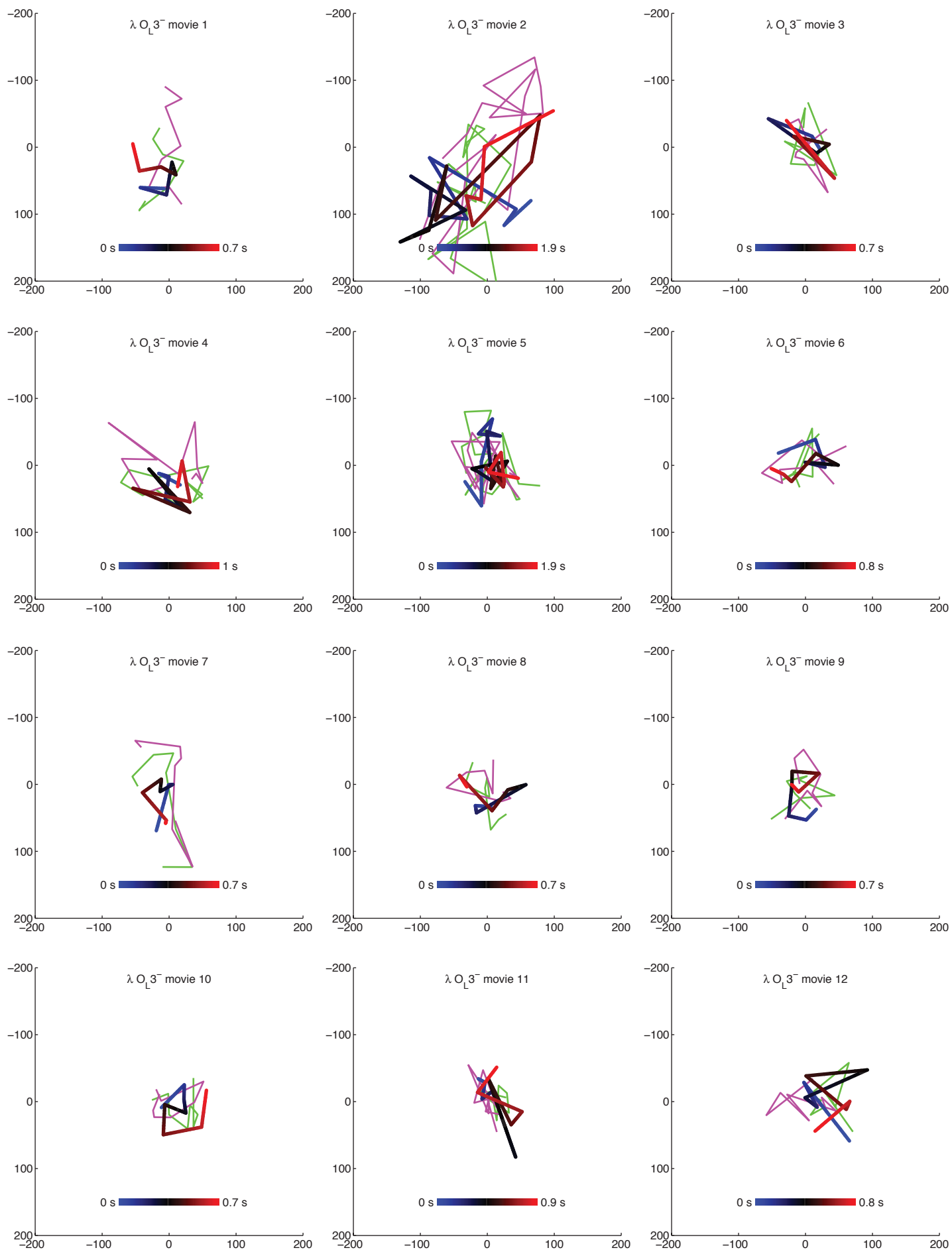

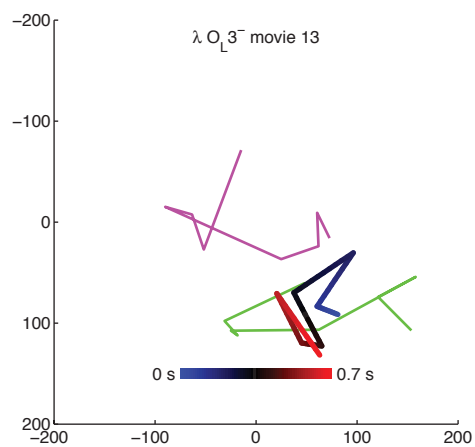

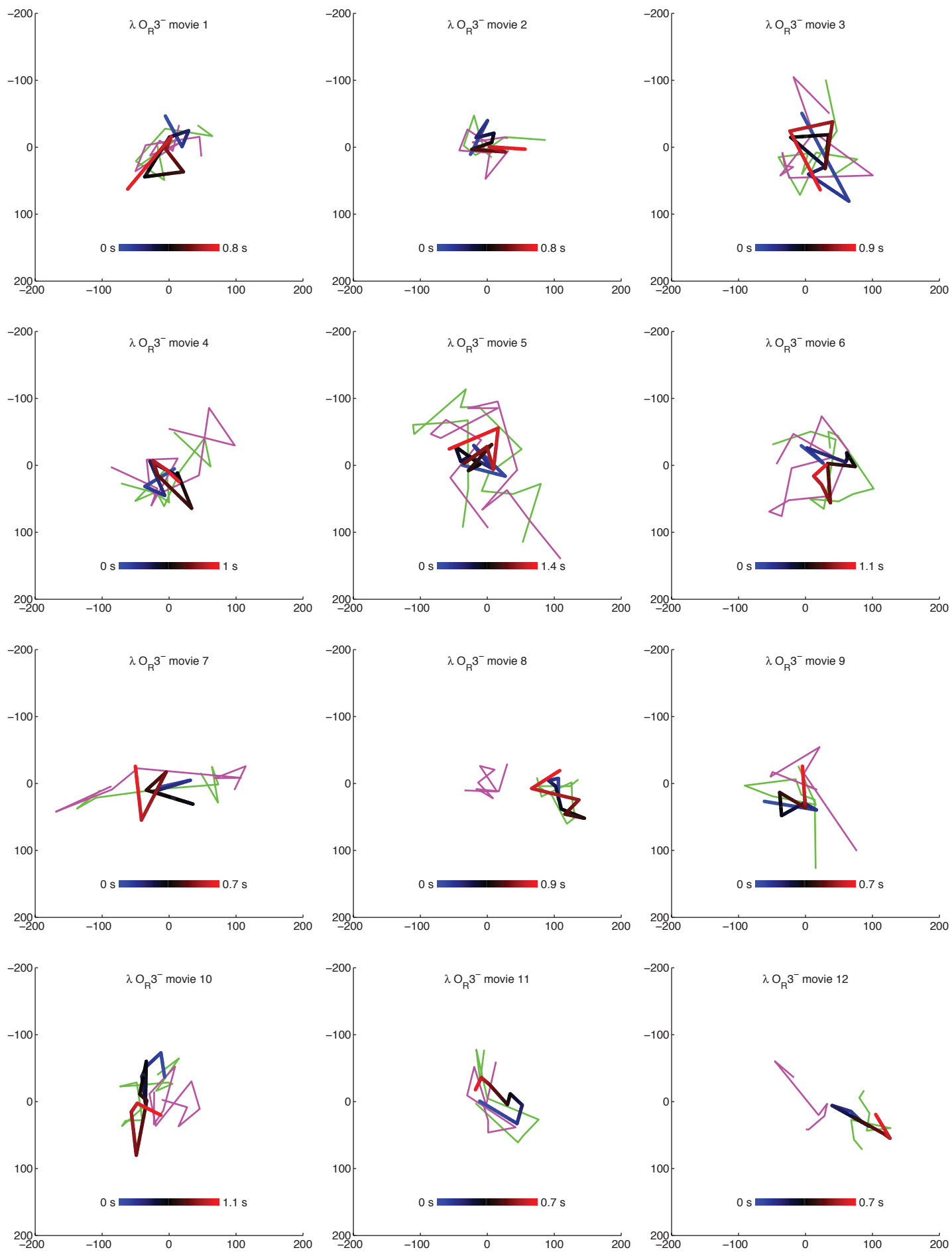

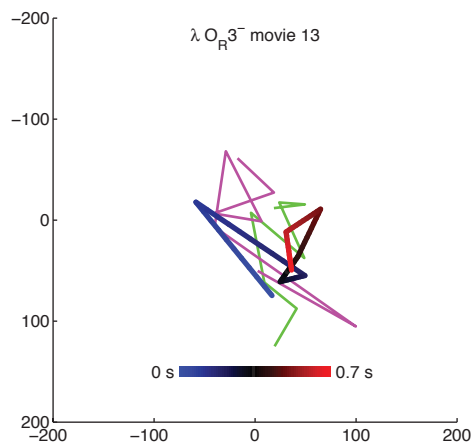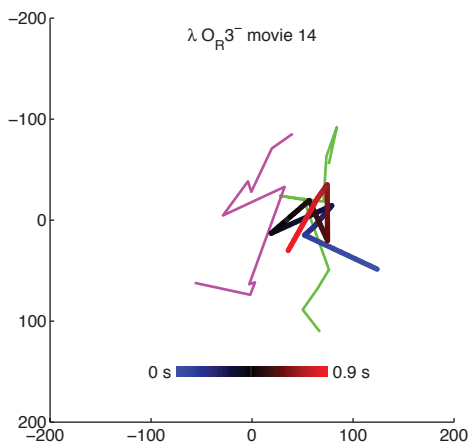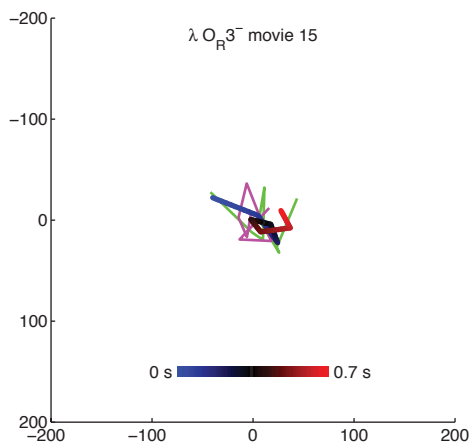

Supplement: Figure S3 — Plots showing trajectories of vectors for all data from all strains for every molecule that was fit in both the EYFP and mCherry images for at least 8 consecutive frames (800 ms). Green and magenta lines are single-color trajectories for TetR-EYFP and LacI-mCherry spots, respectively; the corresponding trajectory with time colored-coded from blue to red is plotted on top at the same length scale. Coordinates are in nm. (PDF) [file pbio.1001591.s003.pdf]

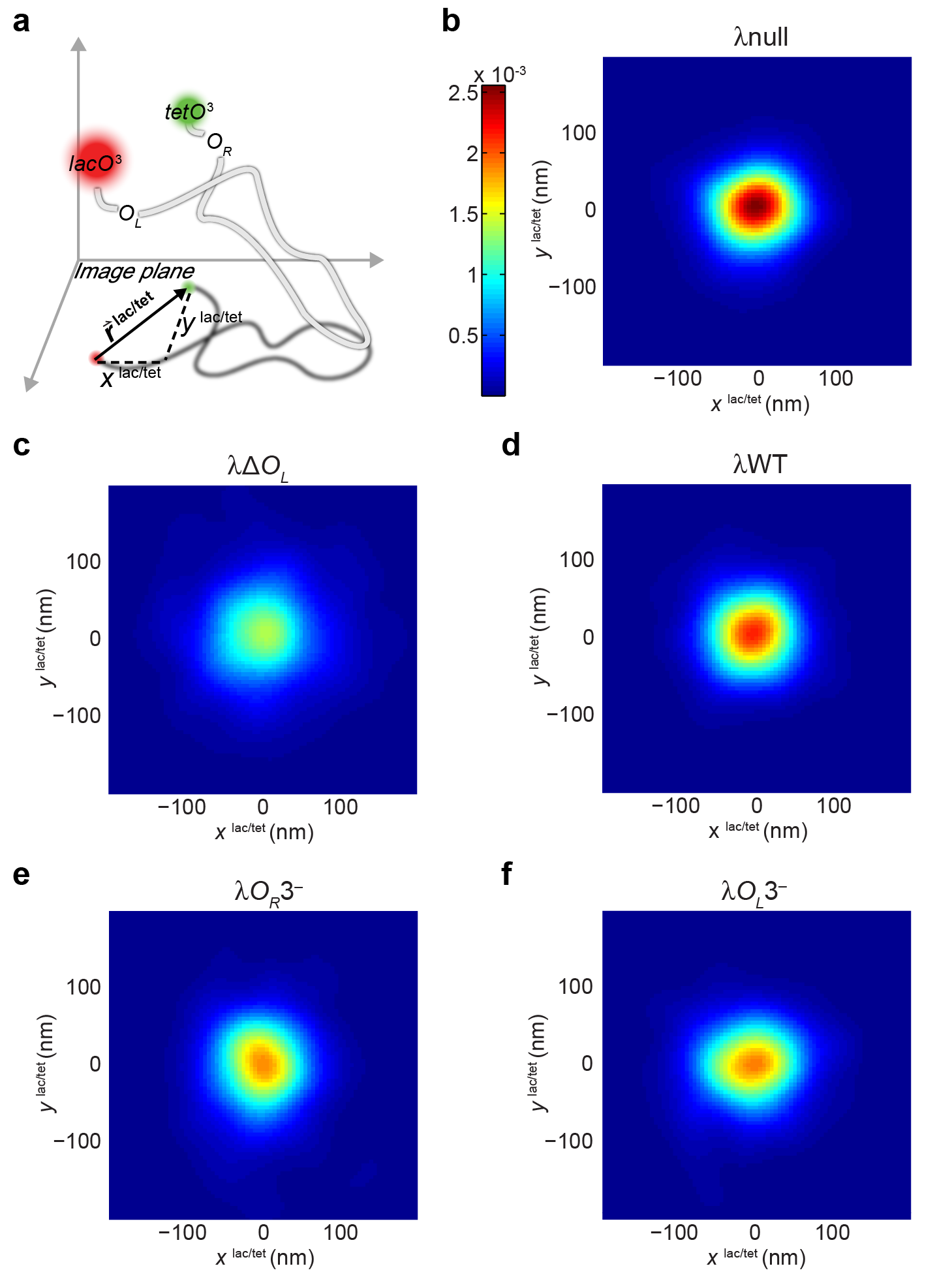

Supplement: Figure S4 — Two-dimensional distributions of the x and y components of vectors. (a) A cartoon describes the calculation of the x and y components of the vector. In the projected image, the vector has two components determined by the arbitrary orientation of the detector. (b–f) Heat maps of the distribution of the x and y components of vectors of each strain. Plots were generated by binning the data for all into 5 nm×5 nm bins. The resulting 2-dimensional distribution was then filtered with a Gaussian kernal (with a width similar to spot-localization precision) to approximate the smoothed distributions. Each image is colored by the probability of the vector falling within a given bin according to the scale bar in (b). (TIF) [file pbio.1001591.s004.tif]

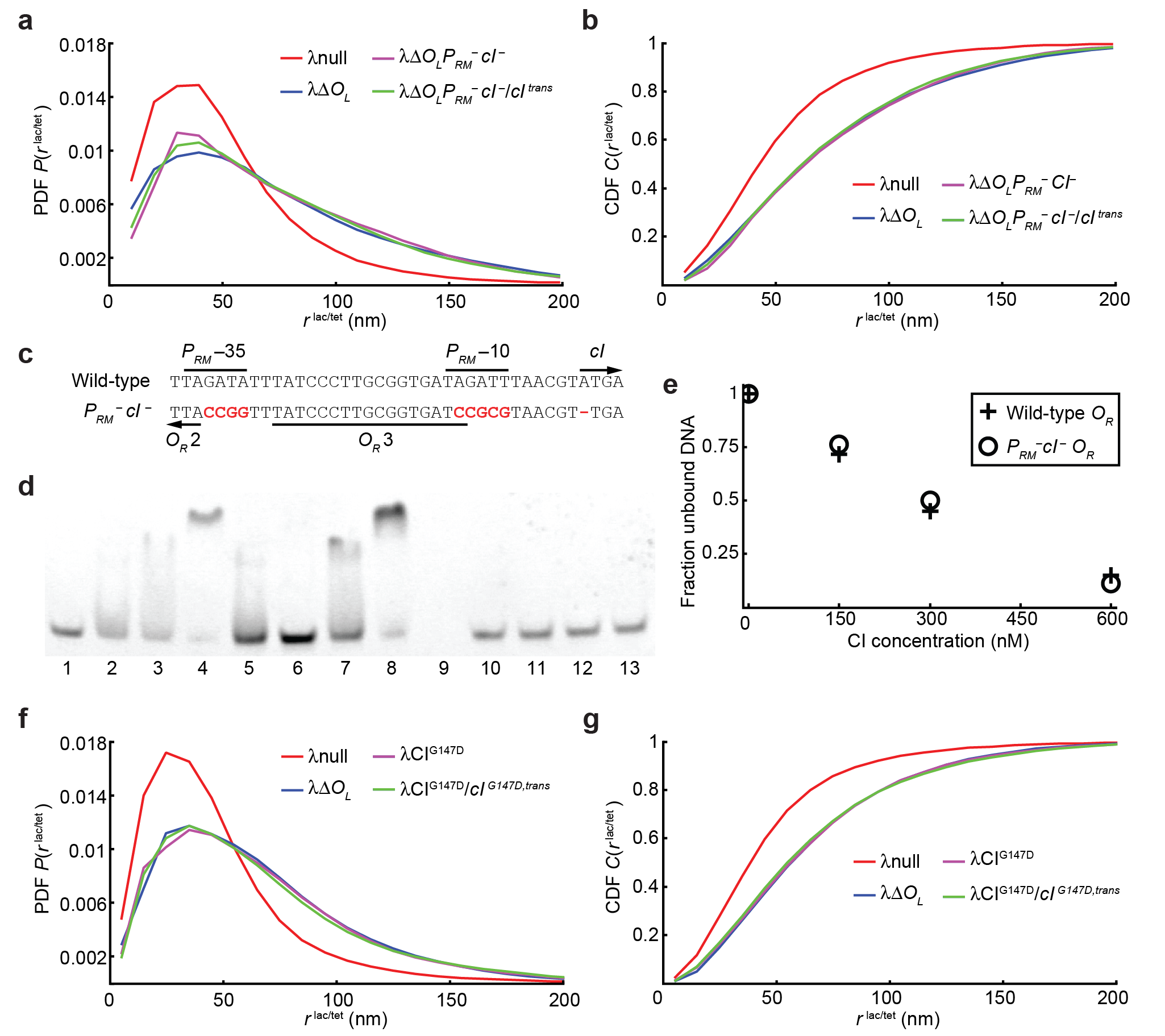

Supplement: Figure S5 — Experiments showing the effects of transcription, nonspecific CI binding and higher-ordered CI oligomer on DNA looping. (a) End-to-end distance () distributions (PDF) for λnull (red), λΔOL (blue), λΔOLPRM−cI− (purple), and λΔOLPRM−cI−/cItrans (green). The PDF is estimated for 10-nm bins. (b) Cumulative density of (CDF) for λnull (red), λΔOL (blue), λΔOLPRM−cI− (purple), and λΔOLPRM−cI−/cItrans (green). The CDF is estimated for 10-nm bins. (c) DNA sequence for the PRM−cI− mutant in comparison to the wild-type sequence. Mutated nucleotides are shown in red. (d) Gel shift assay monitoring the binding of wild-type CI protein. Lane 1–4, CI at concentrations of 0, 150, 300, and 600 nM binding to a 158-bp DNA fragment (20 nM) amplified from the plasmid pZH107 carrying the wild-type PRM DNA sequence. Lane 5–8, CI at concentrations of 150, 0, 300, and 600 nM (note loading order) binding to a 158-bp DNA fragment (20 nM) amplified from the plasmid pACL007 carrying the PRM−cI− sequence. Lane 9: empty. Lane 10–13, CI at concentrations of 0, 150, 300, and 600 nM binding to a 140-bp DNA fragment (20 nM) amplified from the E. coli hns promoter region, which CI does not bind specifically. Reaction mixtures were incubated in a buffer (10 mM Tris pH 8.0, 50 mM KCI, 1 mM MgCl2, 10% glycerol, 100 ug/ml BSA, 1 mM DTT) at room temperature for 10 min. Samples were electrophoresed in Bio-Rad 4–20% Gradient TBE gels (Bio-Rad, Hercules, CA) in a cold room and then stained with Ethidium Bromide for 30 min. (e) Fraction of bound DNA (intensity of low-weight band divided by intensity of lane over background) quantified using NIH ImageJ for the gel shown in (d). (f, g) Distributions of identical in description to those in (a, b) showing strains λnull (red), λΔOL (blue), λG147D (purple), and λG147D/cIG147D,trans (green). (TIF) [file pbio.1001591.s005.tif]

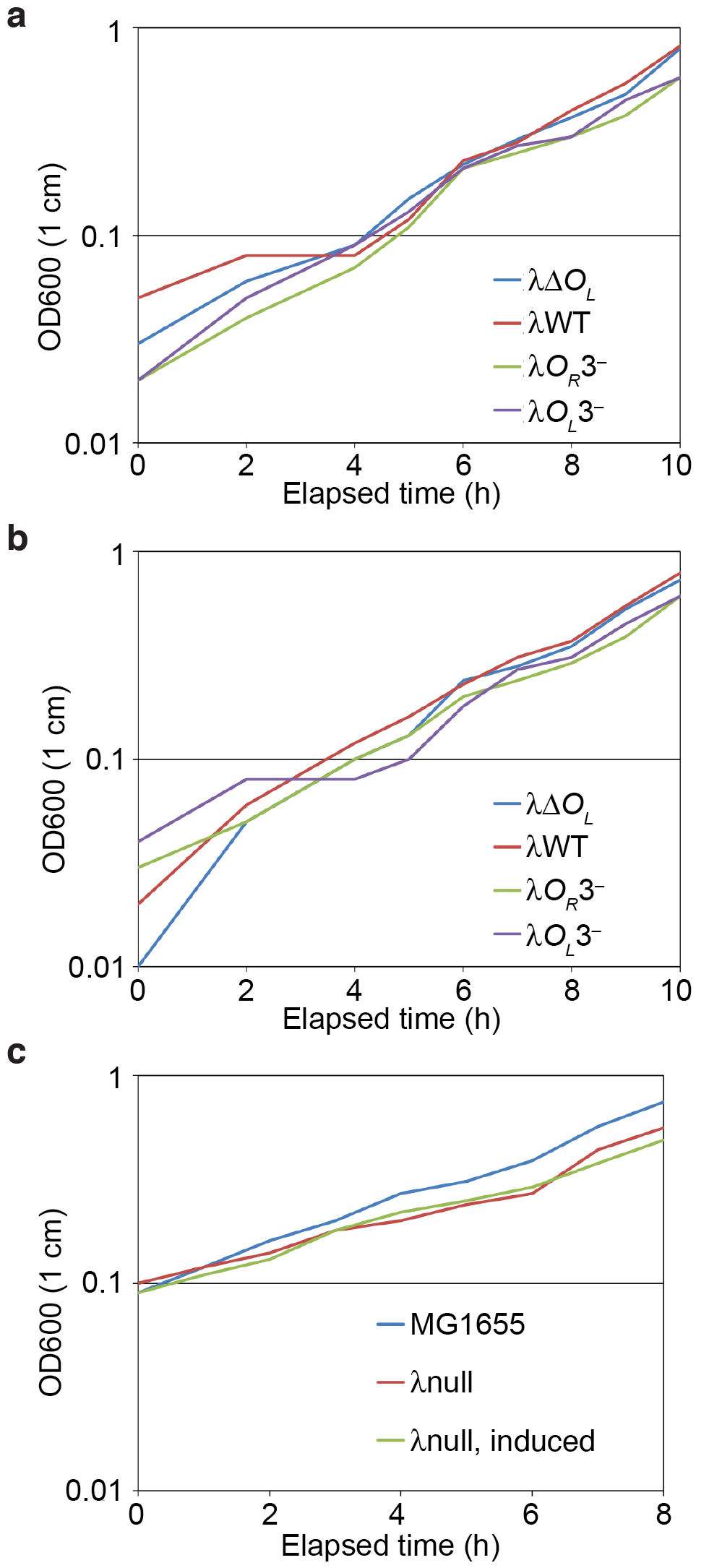

Supplement: Figure S6 — Growth rate comparisons. (a, b) Strains used in thermodynamic modeling were diluted from exponential growth to low optical densities in M9 minimal media supplemented with 0.4% glucose and carbenicillin as described in the main text. OD600 was measured over 10 h of growth for two replicate experiments. Strains are λΔOL (blue), λWT (red), λOR3− (green), and λOL3− (purple). Doubling times calculated using the Microsoft Excel LOGEST function range from 1.7 to 2.5 h. Two independent replicates are shown. (c) Growth rates for the parent E. coli strain MG1655 (blue) were compared to those of the control strain λnull in which the lac operon is replaced with a construct incorporating the lacO3 and tetO3 binding site arrays and which harbors the plasmid pZH102R33Y29 which expresses both TetR-EYFP and LacI-mCherry fluorescent fusion proteins upon arabinose induction. Strains were grown in M9 minimal media supplemented with 0.4% glycerol and λnull was grown in both the absence (red) and presence (green) of 0.3% L-arabinose. Doubling times were 2.7 h for MG1655 and 3.4 and 3.3 h for λnull in the absence and presence of L-arabinose, respectively. (TIF) [file pbio.1001591.s006.tif]
