# Supplementary material for: Transcription-Factor-Mediated DNA Looping Probed by High-Resolution, Single-Molecule Imaging in Live E. coli Cells
Source: PLoS Biol. 2013 Jun 18;11(6):e1001591. doi: 10.1371/journal.pbio.1001591 (PMC3708714; doi:10.1371/journal.pbio.1001591)
Supplement: Table S1 — Looping frequencies were estimated from alternate data sets using either all data or only the data from the first frames (for molecules appearing in more than one sequential frame) and fitting either probability (PDF) or cumulative (CDF) distributions. The first row results for each strain were reported in the main text. (DOCX) [file pbio.1001591.s013.docx]

**Table S1**

| Strain | Sample | Distribution | Looping frequency |
| --- | --- | --- | --- |
| λWT | All frames | CDF | 79 ± 6% |
|  |  | PDF | 78 ± 9% |
|  | First frames | CDF | 81 ± 8% |
|  |  | PDF | 76 ± 11% |
| λ*O_R_*3^–^ | All frames | CDF | 53 ± 7% |
|  |  | PDF | 47 ± 9% |
|  | First frames | CDF | 56 ± 9% |
|  |  | PDF | 57 ± 10% |
| λ*O_L_*3^–^ | All frames | CDF | 60 ± 7% |
|  |  | PDF | 52 ± 9% |
|  | First frames | CDF | 62 ± 8% |
|  |  | PDF | 59 ± 11% |
