# Supplementary material for: Transcription-Factor-Mediated DNA Looping Probed by High-Resolution, Single-Molecule Imaging in Live E. coli Cells
Source: PLoS Biol. 2013 Jun 18;11(6):e1001591. doi: 10.1371/journal.pbio.1001591 (PMC3708714; doi:10.1371/journal.pbio.1001591)
Supplement: Table S2 — States used in thermodynamic modeling. We used free-energy parameters that were described by Dodd et al. [35]. States that will not be populated near lysogenic CI concentrations (e.g., those without OL1 or OL2 bound) are ignored; the reference state () has CI dimers bound to OL1 and OL2. A state with OR free of CI is included to show activation in Figure 5a and b, but does not significantly change fit parameters; because OR1 and OR2 binding is highly cooperative, we do not model states with only one or the other operator bound. The degeneracy term indicates how many microstates exist with identical CI dimer binding patterns and free energies. A particular macrostate may have several microstates that differ in terms of parallel or antiparallel looping configurations or in the identity of binding sites participating in cooperative interactions (either through looping or through adjacent dimers). Here, we also list whether a state is looped (1 for looped; 2 for unlooped) as well as its transcription rate, (0; 1 for ; 2 for ; 3 for ). The free energy of state 2 is called below. (DOCX) [file pbio.1001591.s014.docx]

**Table S2**

| State | Description (operators bound; loop type) | $\Delta G$ (^kcal^/_mol_) | $d$ | Loop | $k$ |
| --- | --- | --- | --- | --- | --- |
| 1 | *O_L_*1, *O_L_*2; unlooped | 0 | 1 | 0 | 1 |
| 2 | *O_L_*1, *O_L_*2, *O_R_*1, *O_R_*2; unlooped | $\Delta G$*O_R_*1 + $\Delta G$*O_R_*2 + $\Delta G$*O_R_*1-*O_R_*2 coop | 1 | 0 | 2 |
| 3 | *O_L_*1, *O_L_*2, *O_R_*1, *O_R_*2; looped octameric | ${\Delta G}_{2}$ + ${\Delta G}_{\text{oct}}$ | 2 | 1 | 3 |
| 4 | *O_L_*1, *O_L_*2, *O_L_*3, *O_R_*1, *O_R_*2; unlooped | ${\Delta G}_{2}$ + $\Delta G$*O_L_*3 | 2 | 0 | 2 |
| 5 | *O_L_*1, *O_L_*2, *O_L_*3, *O_R_*1, *O_R_*2; looped octameric | ${\Delta G}_{2}$ + $\Delta G$*O_L_*3 + ${\Delta G}_{\text{oct}}$ | 4 | 1 | 3 |
| 6 | *O_L_*1, *O_L_*2, *O_R_*1, *O_R_*2, *O_R_*3; unlooped | ${\Delta G}_{2}$ + $\Delta G$*O_R_*3 | 2 | 0 | 0 |
| 7 | *O_L_*1, *O_L_*2, *O_R_*1, *O_R_*2, *O_R_*3; looped octameric | ${\Delta G}_{2}$ + $\Delta G$*O_R_*3 + ${\Delta G}_{\text{oct}}$ | 4 | 1 | 0 |
| 8 | *O_L_*1, *O_L_*2, *O_L_*3, *O_R_*1, *O_R_*2, *O_R_*3; unlooped | ${\Delta G}_{2}$ + $\Delta G$*O_R_*3 + $\Delta G$*O_R_*3 | 4 | 0 | 0 |
| 9 | *O_L_*1, *O_L_*2, *O_L_*3, *O_R_*1, *O_R_*2, *O_R_*3; looped octameric | ${\Delta G}_{2}$ + $\Delta G$*O_R_*3 + $\Delta G$*O_R_*3 + ${\Delta G}_{\text{oct}}$ | 4 | 1 | 0 |
| 10 | *O_L_*1, *O_L_*2, *O_L_*3, *O_R_*1, *O_R_*2, *O_R_*3; looped octameric+tetrameric | ${\Delta G}_{2}$ + $\Delta G$*O_R_*3 + $\Delta G$*O_R_*3 + ${\Delta G}_{\text{oct}}$ + ${\Delta G}_{\text{tet}}$ | 4 | 1 | 0 |
