# Supplementary material for: Transcription-Factor-Mediated DNA Looping Probed by High-Resolution, Single-Molecule Imaging in Live E. coli Cells
Source: PLoS Biol. 2013 Jun 18;11(6):e1001591. doi: 10.1371/journal.pbio.1001591 (PMC3708714; doi:10.1371/journal.pbio.1001591)
Supplement: Table S3 — Thermodynamic model fitting using alternative choices for wild-type CI concentration (expressed here in molecules/cell; in the model, 1 molecule per cell is equivalent to 1.47 nM) and the fraction of CI molecules that are in the form of free dimers. The approximation of a constant free-dimer fraction is reasonable if specifically bound CI dimers (up to 6 dimers composed of 12 monomers) do not make up a large fraction of total CI and if CI concentration is sufficiently high that almost all CI molecules are in dimeric complexes. The free-dimer fractions used here were calculated assuming the absence of specific binding sites using the parameters for nonspecific binding site affinity and concentration estimated by Dodd et al. [35]. Results in the first row are the same as those presented in the main text. (DOCX) [file pbio.1001591.s015.docx]

| **Table S3**  CI molecules per cell | Free-dimer fraction | ${\Delta G}_{\text{oct}}$(^kcal^/_mol_) | ${\Delta G}_{\text{tet}}$(^kcal^/_mol_) | $k_{\text{unlooped}}$(^nM^/_min_) | $k_{\text{looped}}$  (^nM^/_min_) |
| --- | --- | --- | --- | --- | --- |
| 150 | 0.59% | 0.3 | -3.2 | 1.9 | 4.7 |
| 150 | 0.12% | 0.2 | -4.6 | 2.4 | 4.1 |
| 150 | 2.95% | 0.4 | -2.8 | 2.4 | 5.4 |
| 75 | 0.59% | 0.3 | -3.8 | 1 | 2.3 |
| 300 | 0.59% | 0.2 | -2.8 | 3.9 | 9.5 |
