# Supplementary material for: Transcription-Factor-Mediated DNA Looping Probed by High-Resolution, Single-Molecule Imaging in Live E. coli Cells
Source: PLoS Biol. 2013 Jun 18;11(6):e1001591. doi: 10.1371/journal.pbio.1001591 (PMC3708714; doi:10.1371/journal.pbio.1001591)
Supplement: Table S4 — Names of new strains used in this study (as used internally in our lab) and shorthand names used in the main text. (DOCX) [file pbio.1001591.s016.docx]

**Table S4**

| Short-hand name | Strain name |
| --- | --- |
| λnull | ZHX105 |
| λΔ*O_L_* | ZHX106 |
| λWT | ZHX107 |
| λ*O_R_*3^–^ | ZHX107r1 |
| λ*O_L_*3^–^ | ZHX107OL34 |
| λCI^G147D^ | ACL049 |
| λCI^G147D^/*cI^G147D,trans^* | ACL050 |
| λΔ*O_L_P_RM_*^–^*cI*^–^ | ACL007b |
| λΔ*O_L_P_RM_*^–^*cI*^–^/*cI*^trans^ | ACL011 |
