# Supplementary material for: Transcription-Factor-Mediated DNA Looping Probed by High-Resolution, Single-Molecule Imaging in Live E. coli Cells
Source: PLoS Biol. 2013 Jun 18;11(6):e1001591. doi: 10.1371/journal.pbio.1001591 (PMC3708714; doi:10.1371/journal.pbio.1001591)
Supplement: Table S5 — Sequences of oligonucleotide probes for single-molecule fluorescence in situ hybridization (smFISH) experiment. Asterisks indicate probes that do not hybridize specifically with any E. coli sequence. All other probes hybridize nonoverlapping sequence in the cI coding region of the mRNA transcript from the PRM promoter. (DOCX) [file pbio.1001591.s017.docx]

**Table S5**

| GTCTTGACGAAAATCTGCAT* | TGGGATAAGCCAAGTTCATT | CACTGGCTTTTTTGGTTGTG |
| --- | --- | --- |
| TGTTATGGTTTTACCGGTCA* | CATCTTGTCTGCGACAGATT | ACCTCAAGCCAGAATGCAGA |
| TATCGGAAGATTCAACTTCC* | CAACGCCTGACTGCCCCATC | TGCGGTCATGGAATTACCTT |
| TTCGACTTAACGTTGTCGAT* | TTGATGCCATTAAATAAAGC | AGCTTGGCTTGGAGCCTGTT |
| GATACCTTCCTTGTCTTGAA* | GCGGCGTTATAAGCATTTAA | AGAATTAACATTCCGTCAGG |
| TCAATCTTTGTTGATCTGGA* | CTTTGAGAATTTTTGCAAGC | AACAGCCTGCTCAGGGTCAA |
| TCTAGCTGCTTACCGGCAAA* | GGGCTAAATTCTTCAACGCT | TGGCTATGCAGAAATCACCT |
| ATCAGACAGCGTTCTACCGT* | GTAGATTTCTCTGGCGATTG | GTAAACTCATCACCCCCAAG |
| TGGACTCCTTCTGAATGTTG* | TACTAACCGCTTCATACATC | ATCCCTGATCAGTTTCTTGA |
| AGCCTTAGCACAAGATGTAA* | TCACTTCTAAGTGACGGCTG | GTTGTAAAAACACCTGACCG |
| TTTTGTGCTCATACCACCTC* | GAGAAAAAACAGGGTACTCA | ATTGGGTACTGTGGGTTTAG |
| GCTCTTGTGTTAATGGTTTC | TGAGAACATCCCTGCCTGAA | ACAACTCTCATTGCATGGGA |
| AAGGCGACGTGCGTCCTCAA | TGGTAAAGGTTCTAAGCTCA | AGCGATAACTTTCCCCACAA |
| TCTTTTTTTCATAAATTGCT | TACCCATCTCTCCGCATCAC | AAACGTCTCTTCAGGCCACT |
