# Supplementary material for: Transcription-Factor-Mediated DNA Looping Probed by High-Resolution, Single-Molecule Imaging in Live E. coli Cells
Source: PLoS Biol. 2013 Jun 18;11(6):e1001591. doi: 10.1371/journal.pbio.1001591 (PMC3708714; doi:10.1371/journal.pbio.1001591)
Supplement: Table S6 — Measurement statistics for experiment comparing distributions for looped and unlooped control strains to for strains lacking OL and having weakened PRM promoters with and without the overexpression of wild-type CI from a plasmid. Errors for the measurements are all 1 s.e.m. as estimated from 1,000 bootstrapped samples. Note that distributions display small, day-to-day variability between experiments (see Figure S1, this table, Table 2, Table S7), but the trend stays the same for a given set of experiments. (DOCX) [file pbio.1001591.s018.docx]

**Table S6**

| Strain | $r^{\text{lac/tet}}$ measurements | Mean $r^{\text{lac/tet}}$ (nm) | Median $r^{\text{lac/tet}}$ (nm) |
| --- | --- | --- | --- |
| λnull | 951 | 50 ± 1 | 43 ± 1 |
| λΔ*O_L_* | 1503 | 73 ± 1 | 63 ± 1 |
| λΔ*O_L_P_RM_*^–^*cI*^–^ | 856 | 73 ± 1 | 63 ± 2 |
| λΔ*O_L_P_RM_*^-^*cI*^–^/*cI*^trans^ | 867 | 72 ± 1 | 62 ± 2 |
