# Supplementary material for: Transcription-Factor-Mediated DNA Looping Probed by High-Resolution, Single-Molecule Imaging in Live E. coli Cells
Source: PLoS Biol. 2013 Jun 18;11(6):e1001591. doi: 10.1371/journal.pbio.1001591 (PMC3708714; doi:10.1371/journal.pbio.1001591)
Supplement: Table S7 — Measurement statistics for experiment comparing distributions for looped and unlooped control strains to for strains in which CI harbors the G147D mutation with and without the overexpression of CIG147D from a plasmid. Errors for the measurements are all 1 s.e.m. as estimated from 1,000 bootstrapped samples. Note that distributions display small, day-to-day variability between experiments (see Figure S1, this table, Table 2, Table S6), but the trends stays the same for a given set of experiments. (DOCX) [file pbio.1001591.s019.docx]

**Table S7**

| Strain | $r^{\text{lac/tet}}$ measurements | Mean $r^{\text{lac/tet}}$ (nm) | Median $r^{\text{lac/tet}}$ (nm) |
| --- | --- | --- | --- |
| λnull | 1227 | 46 ± 1 | 39 ± 1 |
| λΔ*O_L_* | 1201 | 64 ± 1 | 58 ± 1 |
| λCI^G147D^ | 873 | 65 ± 2 | 58 ± 2 |
| λCI^G147D^/*cI^G147D,trans^* | 1982 | 64 ± 1 | 56 ± 1 |
