# Supplementary material for: Transcription-Factor-Mediated DNA Looping Probed by High-Resolution, Single-Molecule Imaging in Live E. coli Cells
Source: PLoS Biol. 2013 Jun 18;11(6):e1001591. doi: 10.1371/journal.pbio.1001591 (PMC3708714; doi:10.1371/journal.pbio.1001591)
Supplement: Table S8 — CI expression levels measured by smFISH for wild-type phage lambda lysogen JL5392 and additional strains. For strains with replicate experiments (N, number of independent experiments), errors indicate standard deviation. The expression levels were normalized to wild-type units (WTUs) using the λWT strain. (DOCX) [file pbio.1001591.s020.docx]

**Table S8**

| Strain | N | CI expression level (WLU) |
| --- | --- | --- |
| JL5392 | 3 | 1.0 ± 0.5 |
| λΔ*O_L_P_RM_*^–^*cI*^–^ | 2 | 0.2 ± 0.1 |
| λΔ*O_L_P_RM_*^-^*cI*^–^/*cI*^trans^ | 2 | 9.3 ± 2.3 |
| λCI^G147D^ | 1 | 0.2 |
| λCI^G147D^/*cI^G147D,trans^* | 1 | 11.3 |
